# A method for the quantification of phototropic and gravitropic sensitivities of plants combining an original experimental device with model-assisted phenotyping: Exploratory test of the method on three hardwood tree species

**DOI:** 10.1371/journal.pone.0209973

**Published:** 2019-01-25

**Authors:** Catherine Coutand, Boris Adam, Stéphane Ploquin, Bruno Moulia

**Affiliations:** 1 INRA, UR 1115 PSH, Avignon, France; 2 Université Clermont Auvergne, INRA, PIAF, Clermont-Ferrand, France; Universitat Zurich, SWITZERLAND

## Abstract

Perception of inclination in the gravity field and perception of light direction are two important environmental signals implicated in the control of plant shape and habit. However, their quantitative study in light-grown plants remains a challenge. We present a novel method here to determine the sensitivities to gravitropism and phototropism. The method combines: (i) an original experimental device of isotropic light to disentangle gravitropic and phototropic plant responses; and (ii) model-assisted phenotyping using recent models of tropism perception—the *AC* model for gravitropism alone and the *ArC* model for gravitropism combined with phototropism. We first assessed the validity of the *AC* and *ArC* models on poplar, the classical species model for woody plants. We then tested the method on three woody species contrasted by their habit and tolerance to shade: poplar (*Populus* tremula*alba), oak (*Quercus petraea*) and beech (*Fagus sylvatica*). The method was found to be effective to quantitatively discriminate the tested species by their ratio of tropistic sensitivities. The method thus appears as an interesting tool to quantitatively determine tropistic sensitivities, a prerequisite for assessing the role of tropisms in the control of the variability of the habit and/or tolerance to shade of woody species in the future.

## Introduction

Gravitropism and phototropism, the orientation of growth as a function of gravity and light, respectively, are two major processes implicated in the regulation of plant shape [1,2]. Their combination allows aerial organs to forage for light without losing mechanical stability [3]. This requirement is important for erect plants and obviously becomes crucial for trees in which the shoot system is perennial and eventually very large. Moreover, differences in tree shape and the reaction to canopy opening [4] are often interpreted as resulting from different sensitivities to photo- and gravitropism. However, even if several molecular cross-talks between the gravitropic and phototropic pathways have been identified [5], very few quantitative studies have been conducted on the interaction between photo- and gravitropism and none provides numerical values of sensitivities to gravi- and phototropism for trees. This lack of data does not make it possible to compare the contribution of gravi- and phototropism between different tree species and thus prevents further advances in quantifying the contribution of gravi- and phototropism in the control of tree shape (habit) variability.

When plants are subjected to gravitropism, they tend to reach the Gravitropic Set-Point Angle (GSA) [6]. However, the GSA can be modulated by several cues, e.g., light direction [7]. Galland extended the concept of GSA to the cases where both gravi- and phototropism are active: plants tend to reach a photogravitropic equilibrium angle (PEA) determined both by gravity field and light direction [8–9].

Further revisiting of Galland’s experiments by Moulia et al. using the formalized *ArC* model [3] led to the definition of the Photo-Gravitropic Set-Point Angle (PGSA) and its quantitative dependency on photo- and gravitropic sensitivities (but not on the initial tilting angle vs. gravity A_0_, in opposition to initial claims by Galland [8–9]). However, the estimation of the PGSA and, finally, of the photo- and gravitropic sensitivities, have only been conducted so far in one set of experiments using *Avena* coleoptile under continuous light [8,3].

The lack of knowledge about photo*gravi tropic interplay is even worse for trees. To our knowledge, only two studies have investigated the phototropic movements of trees [10–11] and demonstrated that phototropism exists in their radially growing zones (woody stems). The gravitropic movement of tree axes (trunk or branch) has received more interest. Nevertheless, emphasis has been placed on the production of reaction wood, a specialized wood that acts as a motor for the tropistic movement of the woody stems and that has consequences on the quality of wood in the lumber industry [12]. Consequently, no quantitative method is available to quantify tropistic tree sensitivities, which is a prerequisite for assessing the contribution and interaction of photo- and gravitropism in tree shape variability.

**Fig 1:**
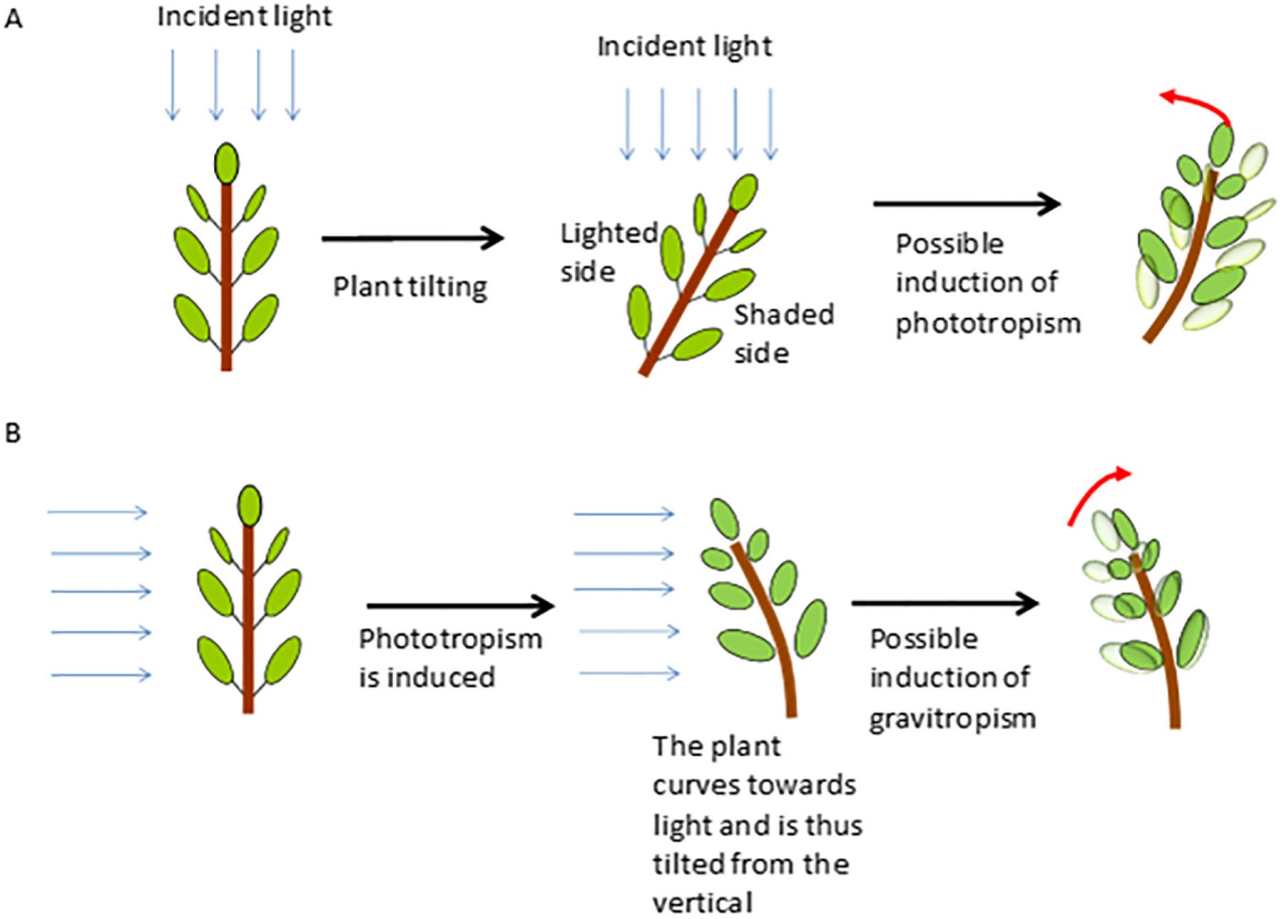
Diagram explaining the interactions between gravitropism and phototropism in classical experiments dedicated to the study of gravitropism and phototropism. A: In classical experiments, a plant is tilted from the vertical in order to study gravitropism. In this case, since the light is coming from the top, one side of the stem is shaded, which can induce a phototropic reaction. B: In classical experiments, a plant is lit unilaterally in order to study phototropism. Since the plant bends towards the unilateral light, the stem is curved and thus tilted, which can induce a possible counteractive movement due to gravitropism.

A quantitative study of the photo*gravi tropistic interactions in the driving of a tropistic movement faces two challenges:

The first challenge is experimental. Indeed, gravitropism and phototropism are not easy to disentangle, at least on Earth ground at 1g. Indeed, as soon as a tree is tilted, one side of the stem is more shaded than the other, which can induce phototropism. Symmetrically, as soon as a vertical tree is lit unilaterally, it bends towards the light, which can trigger gravitropism (Fig 1). An obvious way to address this dilemma is through spatial experiments in microgravity conditions [1] or on 3D clinostats. However, this is not really feasible with trees large enough to undergo significant secondary growth, and devices that would make it possible to fully avoid this dilemma on Earth are lacking.

The second challenge is that formalized modeling is required to analyze the interplay between growth, bending under self-weight and tropisms throughout the tropistic movement [12].

Starting with the pioneering model of Fournier et al. (1994) [13], down to the most recent ones by Coutand et al. [14–15], several models of tree tropisms have been designed [16–17]. All of them have combined: (i) a mechano-biological module of the perception-based driving of the differentiation of reaction wood; and (ii) a biomechanical module of the motor process to compute successive shapes of an axis though the mechanical actions of the differentiation of reaction wood and of the changes in the axis geometry and mass due to growth in girth [12]. This distinction between perception-based driving and motor processes is crucial: indeed, differences in gravitropic sensitivity should not be confused with differences in motor efficiencies (like the one that can be achieved with changes in trunk diameter of wood stiffness [18]). However, most of the research effort has focused on the motor module, taking into account: (i) spatial effects, i.e., asymmetric sections [18], and the heterogeneity of normal and tension wood; and (ii) time effects such as the effects of viscoelasticity and the maturation process [14–15].

Consequently, in all these models, the perception-based driving module is very simple: local inclination from the vertical is perceived all along the trunk and reaction wood is produced in a given cross-section as long as the tree has not reached the vertical position (horizontal cross-section). Models including such a basic perception-based driving module all failed to predict the anticipated decurving of the axis observed on *Pinus pinaster* [13] and on *Populus* [19], as well as in the coleoptile of wheat *Triticum aestivum L*. [20].

Recently, Bastien et al. concentrated on the perception-based driving processes during gravitropism and gravitropism combined with phototropism, and provided two novel models of tropistic driving (*AC* model [20]) and *ArC* model, [3]). They then assessed their models with experiments in which the motor process actuating the movements could be assumed to be non-limiting, mostly in herbs. The *AC* model has been validated on 11 species, constituting a sample of the phylogeny of land angiosperms. This made it possible to simulate the anticipated decurving process observed in pine [13], poplar [19], wheat and other species [20]. However, this validation was generally achieved on the steady-state shape of the plants and mostly for herbaceous species. The only woody plants that were assessed in [20] were two-year-old poplars (data from Coutand et al. [19]), but the assessment of *AC* on poplar was less complete since the achievement of a steady state was not fully assessed. The *ArC* model has only been assessed in one herbaceous species, *Avena sativa* L (and only on the coleoptile of its seedling).

In this work, we propose an original method to compute gravitropic and phototropic sensitivities of trees. Two dedicated experimental devices were designed and built expressly for the development of this method. One, fully original, was dedicated to disentangling gravitropism from phototropism: it makes it possible to tilt young trees in an isotropic light environment, thus triggering the gravitropic response alone. The other is more classical and combines plant tilting and unilateral lightening to study the interaction between gravi- and phototropism. In both set-ups, kinematics of tropistic movements were recorded and fully quantified.

Using these experimental set-ups, we first assessed the validity of the *AC* and *ArC* models for young trees. We then used model-assisted phenotyping with *AC* and *ArC* models to determine gravitropic and phototropic sensitivities of tree seedlings. In this exploratory work, the method was tested on three hardwood species that exhibited different behavior to light and type of growth: poplar (pioneer species, fast growth), oak (hemi-tolerance to shade, rhythmic growth) [21], and beech (high tolerance to shade, slow growth) [22] in order to illustrate the ability of the method to discriminate the three species on the basis of their tropistic sensitivities.

## Material and methods

### Plants

Very young trees at a stage in which the shoot is about 30- to 50-cm-long were chosen because at this stage, the motor process is likely to be non-limiting and gravi- and photogravitropic equilibrium can be reached within a few weeks [13,19].

Poplar: Young poplars (*Populus tremula × alba* cv 717 1B4) were obtained by *in vitro* micropropagation and planted in individual 5-L pots filled with loam after progressive acclimation. Trees were grown in a growth chamber (L/D 16 h/8 h at 24°C/20°C with RH 60 ± 10%). Two months after micropropagation, the poplars were ready to be used in experiments; stems were at least 35-cm high at this stage.

Beech and oak: One-year-old beech and oak seedlings were purchased in a commercial nursery and planted in individual pots filled with 1/3 black peat and 2/3 local “Limagne” soil in autumn 2013 and 2014 and grown in a greenhouse. Watering was ensured on immersion tables. Beech seedlings were protected from direct sun by 55% shade cloth (Puteaux SA, Les Clayes sous Bois, France).

#### Experimental devices to study tropistic responses

We designed and constructed novel experimental devices called “isotropic light spheres” to disentangle gravitropism and phototropism. These spherical growth cabinets allowed young trees to be grown and tilted in an isotropic light environment in such a way that photosynthesis and growth are ensured by light, gravitropism is triggered by tilting the tree, but no phototropic signal is triggered because the lighting is the same from any direction around the tree (isotropy). This device thus made it possible to study the gravitropic response alone during long-term experiments under light conditions.

**Fig 2:**
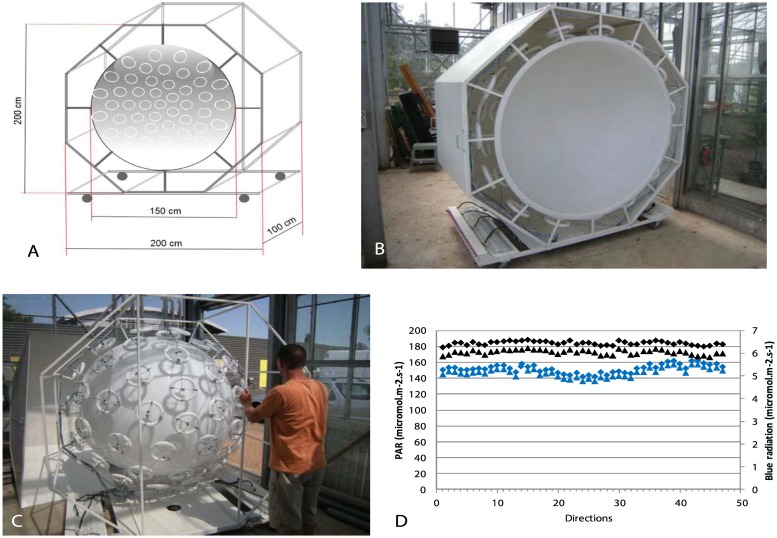
Isotropic light device. A: 3D quoted scheme of the isotropic light device. B: View of one hemisphere showing the turtle pattern of circular neon tubes at the periphery of the white plastic hemisphere. C: View of one hemisphere covered with metallic plates to concentrate the lighting towards the inside of the hemisphere. D: Measurements of PAR and blue radiation in the two spheres in 46 directions with an optic fiber mounted on a robot set in the center of the sphere. Black symbols: PAR; blue symbols: blue radiation.

Two spherical growth cabinets with isotropic light were constructed: each device makes it possible to tilt a young tree in an isotropic light environment. Each sphere was composed of two 1.5-m-diameter hemispheres of transparent polymethyl methacrylate mounted on two specifically designed (INRA, Nancy, France) hexagonal metallic rolling structures (Fig 2A). The inside of each hemisphere was painted white by a bodywork company (Carrosserie David et Fils, Clermont-Ferrand, France) to obtain a homogeneous diffuse light transmission. Each hemisphere was equipped with 46 circular fluorescent tubes (OSRAM, FC 22W/865) placed according to Den Dulk’s ‘TURTLE’ pattern [23]. These neon tubes were chosen because they provided a quality of light that is the closest to sunlight (see spectrum in S1A Fig). The neon tubes were fixed to the outside of the sphere (Fig 2B). In order to maintain the maximum amount of light provided by the neon tubes, reflecting shiny metallic plates were set around each sphere (Fig 2C). Because of the heat produced by the system, a controlled mobile cooling system (Blyss WAP 267EC) was added to each sphere to maintain the temperature at approximately 25°C in the sphere during the lighted periods.

The homogeneity of the emitted radiation of each sphere in terms of blue and photosynthetically-active radiation (PAR) was assessed using a spectrometer (FieldSpec Pro, Analytical Spectral Devices, Inc., Boulder, CO, USA) set on a pan-tilt robot (Biclops P/T, TrackLabs, Webster, TX, USA) to measure radiation in 46 directions. PAR and blue radiation were homogenous within the 46 directions (Fig 2D). PAR was approximately 180 W.m^-2^ and the blue radiation fluence rate was approximately 5 μmol.m^-2^.s^-1^.

**Fig 3:**
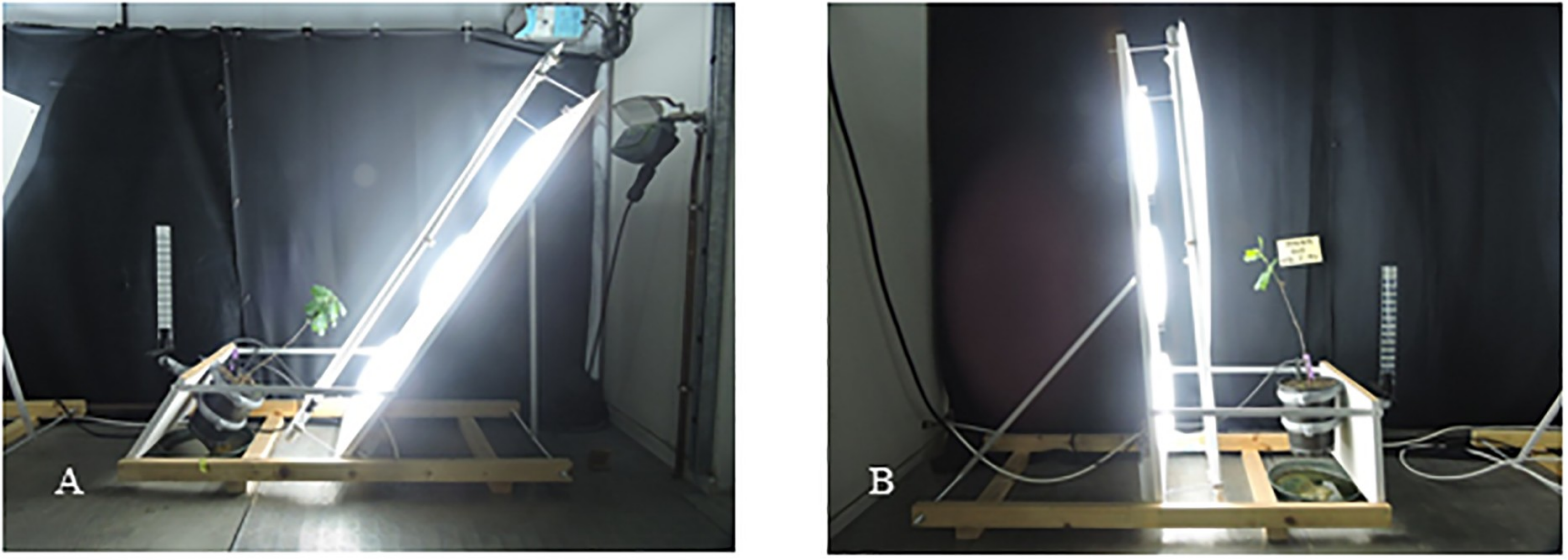
Side views of the anisotropic light device installed in a growth chamber. A: Young oak is installed on the anisotropic light device tilted 35° from the vertical. B: Young oak is installed on the anisotropic light device tilted 5° from the vertical. The walls of the growth chamber were covered with black curtains in order to avoid light reflection.

We also designed lateral light devices to study combined gravitropism and phototropism: The device providing anisotropic lateral light together with plant tilting is based on a pantographic system that makes it possible to choose the same tilting angle for the plant and the board where a light source was mounted (35°, 25°, 15°, 5° (Fig 3A and 3B)). The light source was composed of three circular fluorescent tubes (OSRAM FC 865) set in a row on a white board. These neon tubes were chosen because they provided the quality of light the closest to that of sunlight (the spectrum is available on S1 Fig).

Lighting power could be controlled by changing the neon tubes (55, 40 and 22 watts). A fourth light power of 15W was obtained by setting a neutral filter (216 LEE FILTERS White Diffusion 15FT) in front of 22W neon tubes. The light source came from the lower side of the trees in order to better qualify the tree movements either upward towards the vertical (gravitropism) or downward towards the light source (phototropism). Depending on the size of the plant studied, the three neon tubes could be turned on independently to ensure that the light came only from the lower side of the tilted tree. In our experiments, two neon tubes were switched on for beech and oak. For poplar, since they greatly elongated during their tropistic movements until equilibrium, two, and then very rapidly (two weeks after the beginning of the experiment) three neon tubes were switched on, so that the entire plant remained laterally lit throughout the experiment.

To obtain an exact measurement of light fluence rates reaching the plant stem on the anisotropic light devices, measurements were performed with a ceptometer (AccuPAR LP-80, Decagon Devices) set parallel to the board where light sources were fixed. Measurements were made when the board containing the light sources was tilted at different angles: 0°, 5°, 15°, 25° and 35° from the vertical (S2 Fig). The ceptometer made it possible to measure the photosynthetically-active radiation (PAR). Additional measurements with a spectrophotometer (FieldSpec Pro, Analytical Spectral Devices, Inc., Boulder, CO, USA) allowed us to obtain the spectrum of each type of neon tube (S1 Fig), in particular, blue radiation, the main active one for phototropism. A very similar level of PAR and blue radiation was exhibited by 40W and 50W neon tubes. Spectra values of fluence rates in the PAR and in the blue radiation were computed by integration for every tilting angle and type of neon tube. On the basis of these computations of PAR and blue fluence rates, fluence rates of blue radiation included in the PAR measured by the ceptometer were computed (S1 Table). PAR ranged from 50 to 160 μmol.m^-2^.s^-1^ and blue radiation ranged from 6 to 20 μmol.m^-2^.s^-1^.

PAR values and blue radiation values are given in S1 Table.

To provide full control of the temperature and humidity, the anisotropic light devices were placed in growth chambers. The walls of the growth chambers were covered with black curtains to avoid reflection of light on the walls. Four anisotropic light devices could be installed in a growth chamber at the same time and two similar growth chambers were available at the same time, making it possible to experiment on eight plants simultaneously.

We monitored growth and tropistic movements. Stem diameters were measured weekly using a caliper. Secondary growth was expressed as the average daily diameter growth rate measured at 10 cm from the stem base. Tropistic movements were recorded every 2–3 days by photographs until equilibrium was reached (no detectable movement for two weeks). In each treatment, a picture of the main stem was taken with a digital camera (CASIO, Exilim EX-F1). In order to obtain the scale within each picture, a gradual test card was fixed on each tilting device in the plane of the plant and set at the vertical. For each picture, particular care was taken to align the vertical bars of the grid of the camera screen with the vertical position using the gradual test card. The shape of the main stem on each picture was recorded by digitizing with ImageJ software (NIH) in order to obtain the coordinates (x,y) of each point along the stem. Sets of digitized points give the shape of each seedling stem. Two successive digitized points were approximately 0.5 cm apart.

A preliminary work was performed to assess experiment length: one young tree was tilted 35° on the isotropic light device and a very similar one was tilted on the anisotropic light device. Stem movements were then recorded until a steady-state shape was reached (no movement detected for two weeks).

In poplar seedlings, the fast growing species, the time of the experiment necessary to reach the steady state was approximately four weeks. In oak and beech, which are classified as slow growing species, the required time was found to be about two months.

#### Treatments

Study of gravitropism alone: Young poplar, beech and oak were tilted in an isotropic light environment (one tree per device in each experiment) in controlled conditions (25°C/22°C, 16 h L/8 h D). Two replicates were performed per species.

Study of combined gravitropism and phototropism: Young poplar, beech and oak were tilted in an anisotropic light environment (one tree per anisotropic light device in each experiment) in controlled conditions (25°C/22°C, 16 h L/8 h D). Each species was submitted to a cross-plot design of gravi- and phototropic stimuli (treatments) with four tilting angles (5°, 15°, 25° and 35°) and four neon tube powers (15W, 22W, 40W and 55W). Because this study focused on methodological feasibility assessment and the experiments were long, all 16 treatments were conducted in only one replicate. Nevertheless, the four tilting angles under the 40W neon lighting were replicated for poplar and oak, but not for beech due to experimental hazards. For this last species, only the 35° tilting treatment (which induced photogravitropic movement; see Results section) could be repeated once with 40W neon tubes. Note additionally that the 55W neon tubes provided values of PAR and blue radiation similar to those of the 40W neon tubes (so that the two treatments are in fact almost replicates).

#### Anatomical study

For tilted beech seedlings, detection of tension wood was performed following a classical double-staining procedure with safranin and astra blue (dx.doi.org/10.17504/protocols.io.rdmd246).

### *AC* and *ArC* models used to determine tropistic sensitivities

#### AC model (perception of gravitropism)

In the *AC* model of perception-based driving, the gravitropic movement was found to be directly driven by the perception of both local inclination (gravitropism) and of curvature induced by the tropistic movement (proprioception) [20]. More accurately, during the gravitropic movement, at any time *t*, the curvature rate of the stem located at a spatial position *s* along the stem is predicted to be driven by the sum of perceptions of local inclination (*A*_(*s*,*t*)_) and of the curvature (*C*_(*s*,*t*)_):

$$\frac{dC_{\left(s,t\right)}}{dt}=\beta A_{\left(s,t\right)}+\gamma C_{\left(s,t\right)}$$ (1)

where parameters β and γ are the sensitivity to gravitropism and sensitivity to proprioception, respectively.

Note that if the model is linear in its parameters of gravi- and proprioceptive sensitivities, the differential equation is not linear because A is the spatial integral of C.

This study also demonstrated that the gravitropic equilibrium can only be reached through the balance between graviperception and proprioception. Indeed, the model predicts the angle of the stem from the vertical, at a location s along the stem at the steady state (GSA), as:

$$A_{\left(s,t\right)}=A_{0}exp^{-\frac{\beta s}{\gamma }}$$ (2)

Dimensional analysis of Eq (1) leads to the definition of the dimensionless parameter B, called the Balance number:

$$B=\frac{\beta }{\gamma }L_{0}$$ (3)

where *L*_*0*_ is the initial length of the part of the stem that is active in the tropistic motion.

B is thus the ratio of the gravi- and proprioceptive sensitivities, scaled to the active size of the plant (the size of the zone of the stem that is both sensing and actuating the gravitropism). B is an essential parameter controlling both the final shape of the shoot and its overall gravitropic dynamics [20]. For the same trunk length, the shape of the trunk at gravitropic equilibrium displays more or less concentrated and intense curving depending on the ratio of sensitivity to gravitropism and sensitivity to proprioception. On the basis of Eq 2, the value of B can be estimated through the fit of the field of local inclination along the trunk at gravitropic equilibrium by an exponential, together with the length of the trunk. Note that B can also be determined by fitting Eq 1 of the *AC* model during the tropistic movement, but according to [20], estimating B on the steady-state shape was easier because angle measurements are much less noisy than those of curvature that involve a spatial derivative of the angle, thus enhancing the noise. However, such measurements and estimations of B for a pure gravitropic stimulus have only been conducted in primary growing zones of herbaceous plants grown in the dark [20].

#### *ArC* model (perception of combined gravitropism and phototropism)

Bastien et al. extended the *AC* model to include phototropism, leading to the *ArC* model [3]. The *ArC* model maintains the same characteristics of the *AC* model for gravitropism but also integrates the perception of light direction (phototropism) by including a sensitivity to the angle (App) between the apex direction and the light direction. At photogravitropic equilibrium, the top of the plant is set at the PhotoGravitropic Setpoint Angle (PGSA), also designated Ar (for resultant angle) for convenience in the *ArC* model.

In the *ArC* model, during the photogravitropic movement, at any time t and position s along the stem, the curvature rate of the stem is driven by the sum of three perceptions: perception of local inclination (gravitropism), perception of curvature (proprioception) and perception of the angle between the apex and the light direction (phototropism). The model has three parameters: the first two are shared with the *AC* model (β—the sensitivity to gravitropism-; γ—the sensitivity to proprioception-) and the third,ν, is the sensitivity to phototropism. The equation of the model is the following:

$$\frac{dC_{\left(s,t\right)}}{dt}=\beta A_{\left(s,t\right)}+\gamma C_{\left(s,t\right)}+v\left(A_{\left(L,t\right)}-A_{p}\right)$$ (4)

where L is total stem length, *A(s*,*t)* is the local inclination, *C(s*,*t)* is the curvature, *A(L*,*t)* is the inclination of the tip of the stem (s = L), and *Ap* is the inclination of light from the vertical. *A(L*,*t)*—*Ap* is thus the angle between the apex and the light direction (*App*).

Dimensional analysis of the equations of the *ArC* model leads to two dimensionless numbers, B and M:

$$B=\frac{\beta }{\gamma }L_{0}$$ (5)

and

$$M=\frac{\beta }{\nu }$$ (6)

Thus, the *ArC* model adds a second character measured by the dimensionless number M to the balance number B, the “Motion-pointing number”, which is the ratio between gravi- and phototropic sensitivities. It was in fact shown that for a given angle of incoming light from the vertical (Ap), the Photo-Gravitropic Setpoint Angle Ar only depends on M:

$$\frac{Ar}{Ap}=\frac{1}{1+M}$$ (7)

Together B and M fully control the tropistic dynamics and the final steady-state shape of the shoot at photo-gravitropic equilibrium. They are thus sufficient for a complete quantitative phenotyping of the sensitivity balance involved in driving the tropistic movement resulting from the interaction between photo- and gravitropism (also involving proprioception).

Similarly to the estimation of B using the *AC* model, M can be determined using the *ArC* model equations (model–assisted phenotyping) in two ways: (i) by statistical fitting of the model Eq (4) on an experimental dataset recording the photogravitropic movement vs. time (curvature rate, local inclination, curvature, angle of the apex with incident light) to estimate the β and ν parameter values; and (ii) by computing the ratio Ar/Ap from the measurement of Ar on the photograph of the stem at PGSA and the direction of the incoming light from the vertical (Ap).

Note that in addition to M, B can also be determined using the equation of the *ArC* model during tropistic movement, knowing β and γ parameter values. Bastien et al. [3] did not retain this approach for two reasons: (i) parameter estimates on the curvature dynamics are noisier; and (ii) estimating the three sensitivities at once using a single equation and a single experiment is prone to statistical biases linked to parameter correlations and compensations. Therefore, they suggested a two-step phenotyping: phenotyping of B using the *AC* model on purely gravitropic experiments, and then phenotyping of M using the *ArC* model on photo-gravitropic experiments.

Note finally that the amount of incoming light intensity can affect the value of M and, in the *ArC* model, the function can be either logarithmic (Fechner’s law [8–9]) or a power function (Steven’s law [3]).

The *ArC* model was assessed [3] using the detailed experimental data produced by Galland on *Avena* coleoptiles [8–9]. It was shown that Ar is determined simply by the relative sensitivities to gravitropism and phototropism through the number M and the light direction from the vertical (Ap) but not by the initial tilting angle of the tree from the vertical, as predicted by the *ArC* model and contrary to the initial claim by Galland [3]. Additionally, photogravitropic equilibrium Ar was found to be dependent on the light fluence rate following a power law function, at least in *Avena* coleoptiles. However, the *ArC* model has not yet been assessed on data from woody species.

### Statistical assessment of the *AC* and *ArC* models with data of tropistic movements in poplar

The *AC* and *ArC* models were tested on poplar data to estimate values of their parameters of sensitivities to gravitropism (β), proprioception (γ) (*AC* model), gravitropism (β), proprioception (γ) and sensitivity to light direction (ν) (*ArC* model). To do this, the rate of curvature was considered as the variable to be explained, and local inclination and curvature were the explicative variables for the *AC* model; inclination, curvature, and angle between the apex and the light were the explicative variables for the *ArC* model. *AC* and *ArC* models are linear in their parameters so they were tested through multivariate linear regression (LinearModel.fit procedure of Matlab). β and γ parameters were computed for trees for which only gravitropism was triggered, and β,γ and ν were determined for each tree for which both gravi- and phototropism were triggered. To assess the values of parameters of the *AC* and *ArC* models, statistics with type III errors were used because predictive variables could be correlated. Indeed, the curvature field is obtained from spatial derivatives of the local inclination field and incident light is correlated with the inclination of the pot so that the angle between the apex and the incident light might be correlated with the local inclination.

#### Input data for running a statistical multivariate linear fitting of the *AC* model (gravitropism only)

On the basis of datasets of digitized points of stem shapes, the curvature field was computed for each shape of each stem following the Matlab procedure detailed in [19]. Briefly, each stem shape was first fitted by a smoothing spline. Then, first and second derivatives of the spline function (*P(x)*) were calculated. The local curvature at any material point (i) located at a distance (s) from the stem base was then computed by the following formula:

$$C_{i}=\frac{d^{2}P(x_{i})/dx^{2}}{{\left(1+{\left(dP(x_{i})/dx\right)}^{2}\right)}^{3/2}}$$ (8)

The local inclination field of each shape of each stem was computed by spatial integration of the curvature field and the initial tilting angle. Note that in this study, 0° refers to the vertical position and angles are counted positively in the counter-clock direction.

Both curvature and local inclination are the explicative variables of the linear regression analysis.

The fields of curvature rate along each stem were computed from computed fields of curvature and the time elapsed between two successive photos. The curvature rate was the variable to be explained in the linear regression analysis. Computations were made considering the displacement of material points due to elongation in the upper part of the stem during the tropistic movement [24] using REGR measurements, via the classical technique of ink marks [25–26] on another set of seedlings.

#### Input data for running a statistical multivariate linear fitting of the *ArC* model (combined gravitropism and phototropism)

In addition to the input data for the *AC* model, the inclination at the tip of each shape of each tree was extracted from the field of local inclination and the angle (App) between the apex (local inclination at the tip of each stem shape), and the direction of light from the vertical (Ap) was computed (S3 Fig). Since the light source was fixed on a board that was tilted at the same angle as the angle imposed on the plant pot, the angle of light with the vertical (Ap) is directly computed from the inclination imposed to the plant pot (α) by: *A*_*p*_ = −90 + *α*.

For poplar, M was then computed from values of sensitivities β and ν obtained (Eq 4) with a statistical multivariate linear model run with data from the curvature rate kinetics (variable to be explained) and local inclination, curvature and angle between the apex and the light direction (explicative variables).

Note that the values of parameters given by statistics also made it possible to compute the parameter B, following the same procedure as with gravitropism data alone, using Eq 3.

The regressions analyses provided satisfactory results concerning the statistical significance of the models so that we computed parameter B for every tree using the *AC* model, and parameters B and M using the *ArC* model for most of the trees tested for combined gravi- and phototropism.

### Computation of B and M parameters from the final shape with *AC* and *ArC* model equations at steady state

#### Computation of parameter B from the equation of the *AC* model at steady state

For computing parameter B, the local inclination field along each stem vs. the curvilinear abscissa (position along the stem from the stem base) at gravitropic equilibrium was fitted with an exponential function following Eq 2. The value of parameter a of the fit provided the inclination at the stem base, and the value of b of the fit gave the ratio β/γ. The value of B was then obtained using Eq 3. Note that this way to determine B could only be used with data obtained using the isotropic light device (gravitropism alone).

#### Computation of M parameters from the equation of the *ArC* model at steady state

To determine M, Ar was measured at photogravitropic equilibrium (PGSA) in the combined photo- and gravitropism experiments. Ar was measured at the top of the stem (below the annual shoot for beech). The value of M was then computed for each seedling using Eq 7

### Effect of light intensity on M parameter values

To assess the dependency of M vs. blue light intensity, M was plotted as a function of the blue light fluence rate (the relevant waveband for phototropism) and either fitted with a power function (Steven’s law), or fitted with a logarithmic function (Fechner’s law).

### Comparison of the estimates of B and M parameters from kinetics and from final shape at steady state

For poplars, B and M parameters were computed by two means:

1. The values of parameters were first computed from the values of sensitivities (β,γ,ν) obtained by linear regression using Eqs 3, 4 and 7 (model parameter method), only when the *ArC* model parameters were statistically significant at the level of 5% and when β and γ and when β and ν had the same signs (otherwise leading to negative values of B and M, respectively).
2. The values of parameters B and M were also computed from the stem shapes at gravi- and photogravitropic equilibrium (“final shape method”)

Values of both B and M obtained from the two methods (“final shape method” or “model parameter method”) were compared. For B, the two methods were compared by a pairwise t test for both the radially growing zone and for the whole stem, thereby providing a complete test of the *AC* model.

## Results

### Growth and characteristics of tropistic movements of each species

#### Growth

All light conditions tested in this study were sufficient to ensure growth in girth for the three species studied (S2 Table). As expected, poplar exhibited the highest growth rate, which was about five times more than for oak and beech. Oak grew slightly faster than beech.

#### Tropistic movements

**Fig 4:**
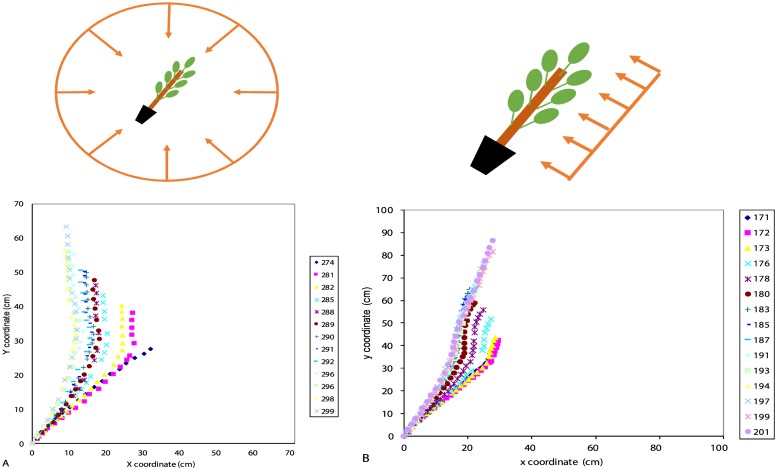
Successive shapes of poplar stems subjected to tilting in an isotropic light environment or in an anisotropic light environment. A: Isotropic light environment: the stem tends to be vertical at steady state. B: Anisotropic light environment: the stem remains inclined at steady state.

Poplar: When subjected to tilting in an isotropic environment (gravitropism alone), the young poplar stem uprighted itself until a large part of the stem reached the vertical position, leading to a steady state (Fig 4A). When they were subjected to tilting in an anisotropic environment, the stem uprighted itself and then stabilized at an angle (Ar) different from the vertical (Fig 4B). The photogravitropic movement was continuous until equilibrium (PGSA).

**Fig 5:**
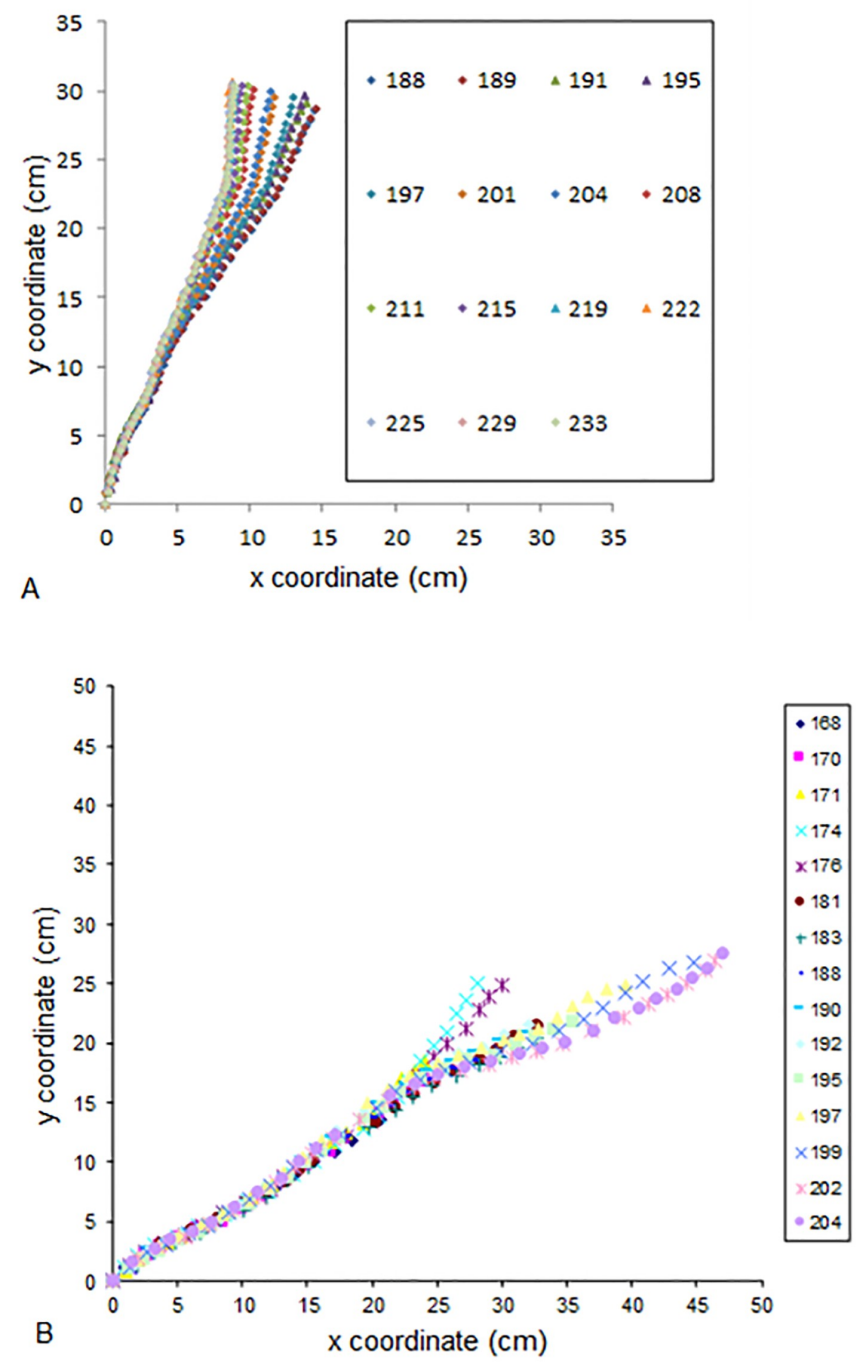
Examples of tropistic movement of oak seedlings subjected to tilting in an isotropic or anisotropic light environment. A: Case of oak seedling subjected to tilting in an isotropic environment: the stem stabilized with the top of the stem back to the vertical. B: Case of oak seedling subjected to tilting in an anisotropic light environment and for which an annual shoot elongated during treatment: the stem stabilized towards light. C: Case of oak seedling subjected to tilting in an anisotropic light environment and for which no annual shoot elongated during treatment: the stem uprighted itself and stabilized around the vertical position.

Oak: When oak trees are subjected to tilting in an isotropic light environment (gravitropism alone), stems stabilized with the top of the stem back to the vertical position (Fig 5A). When the annual shoot elongated while the seedling was tilted in an anisotropic environment, the stem bent towards the light (Fig 5B). However, when no annual shoot elongated (case of three seedlings among twenty seedlings) while the seedling was tilted in an anisotropic light environment, the stem uprighted itself as if the seedling was subjected to tilting alone (Fig 5C). Since our focus in this study was on photo*gravitropic interactions (and not on the location of phototropic sensing), these latter cases were not considered in the following.

**Fig 6:**
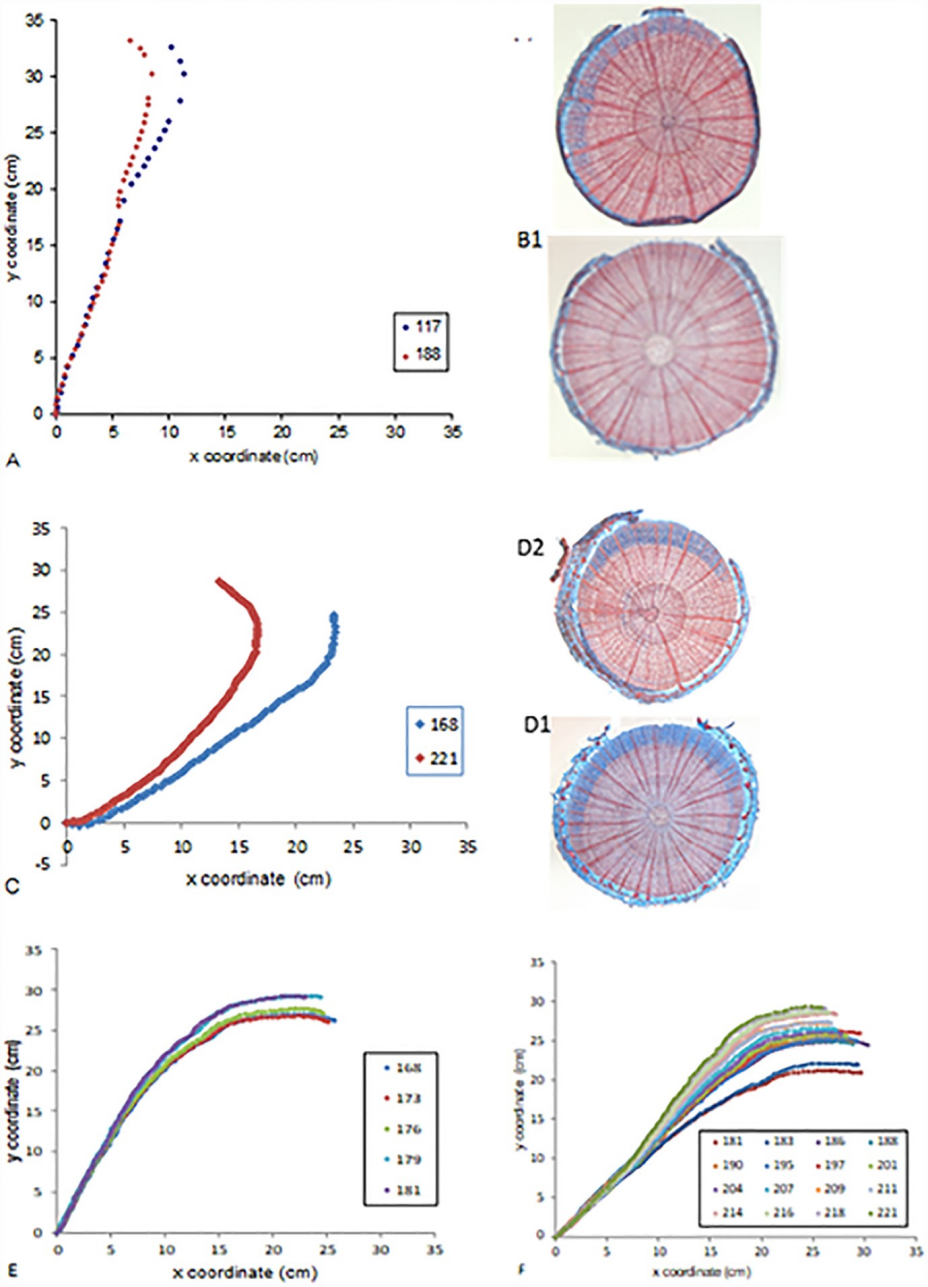
Shapes of beech trees just after tilting and at equilibrium, in an isotropic light environment. A: Pots were tilted 20° from the vertical. No movement could be detected except at the top of the stem (annual shoot), which was tilted more than 20°. B: Photos of anatomical double-stained transversal sections of the stem: B1: Section cut between 0 and 10 cm from the stem base: no reaction wood can be detected; B2: Section cut in the upper part of the stem below the annual shoot: a sector of reaction wood can be detected (crescent stained blue). C: Pots were titled 45° from the vertical. Tree exhibited a visible upward movement. D: Photo of anatomical double-stained transversal sections of the stem. D1: Section cut between 0 and 10 cm from the stem base: a sector of reaction wood can be detected. D2: Section cut in the upper part of the stem below the annual shoot: a sector of reaction wood can be detected. E: Pot was tilted 20° from the vertical for two weeks. The shapes of the stem during the two weeks of tilting are represented; dates are indicated on the graph (Julian days). Almost no movement can be detected. F: The tree was first tilting 20° from the vertical (shown on E) and then the tilting was increased to 45°. Stem shapes until gravitropic equilibrium are presented at the different dates indicated on the graph. The stem uprighted itself.

**Fig 7:**
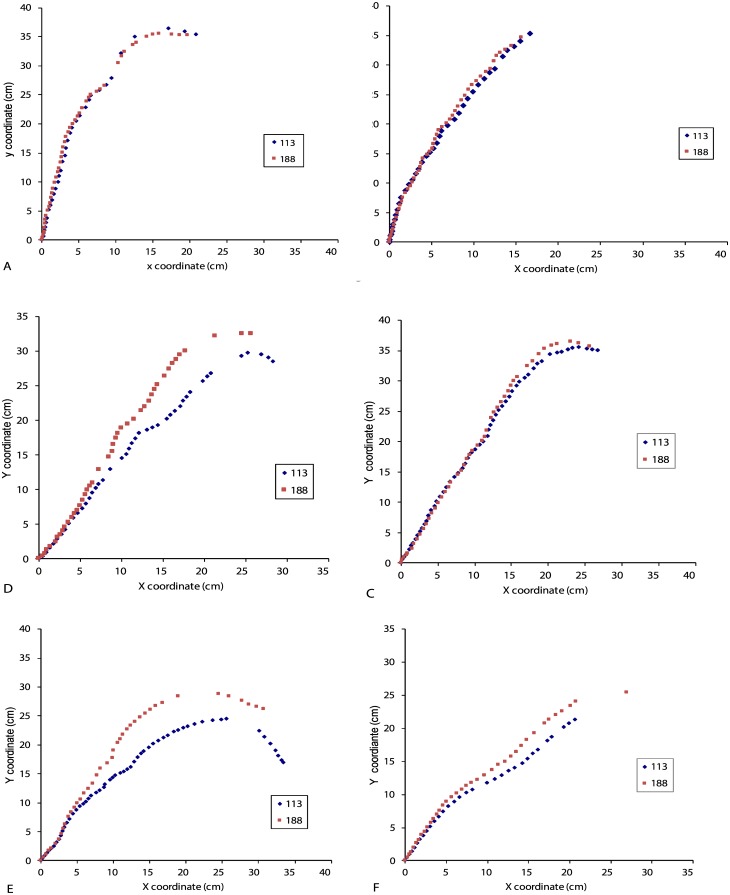
Beech seedling shapes just after tilting and at photogravitropic equilibrium in an anisotropic light environment, for different values of initial tilting. Initial shape (blue symbols) and shape at photogravitropic equilibrium (red symbols) obtained by photo digitizing with Image J. Dates of photos are indicated on the graph (Julian days). A, B: Pots were tilted 5° and 15° from the vertical, respectively. No movement could be detected. C, D: Pots were tilted 25° from the vertical. Tree exhibited no movement (C), or movement (D). E, F: Pots were tilted 35° from the vertical. Trees exhibited a visible movement spread throughout the stem length.

Beech: When beech seedlings were tilted at various angles from the vertical in an isotropic light environment, two cases were observed. When the tilting angle was smaller than 20°, no movement was observed. When the tilting angle was larger than 25°, the stem (except for the annual shoot) uprighted but stabilized at an angle of about 20° from the vertical (Fig 6). When beech seedlings were tilted at various angles from the vertical in an anisotropic light environment, two cases were observed. When the tilting angle was smaller than 20°, no movement was observed; when the tilting angle was larger than 20°, the stem (except for the annual shoot) uprighted but stabilized at an angle larger than 20° from the vertical (Fig 7). In the following, we focused on the cases where tropistic movements could be observed.

### Assessment of the *AC* and *ArC* models with the poplar datasets

#### *AC* model

β, γ parameter values of the *AC* model were determined by linear regression on tropistic kinematics: For each of the two poplar seedlings, results and statistics of the regression analysis are given in S3 Table.

For the radially growing zone, the linear model was statistically highly significant, but it only explained from 20% to 31% of the variability of the curvature rate. Values of both parameters β and γ were highly significant. B values were then obtained for stem 1(B = 2.4) and for stem 2 (B = 2.98) (Table 1, “model parameter method” column).

**Table 1:** Values of gravitropic (β) and proprioceptive (γ) sensitivities (SI unit) and B determined with *AC* model phenotyping with the “parameter model method” and the “final shape method” in the radially growing zone of the stem (i.e., stem with the exception of the annual shoot).

	Seedling 1		Seedling 2	
	Parameter method	Final shape method	Parameter method	Final shape method
β	-5.8484e-06	-	-2.2874e-05	-
γ	-8.5109e-07	-	-2.8895e-06	-
β/γ	6.872	6.321	9.946	7.527
L0 (m)	0.35	0.35	0.3	0.3
B	2.4	2.21	2.98	2.26

Considering regression on the whole stem, the model was also highly significant. β and γ were also computed from results of linear regression with data on the whole stems (annual shoot included). This led to B = 2.12 for stem 1, and B = 3.05 for stem 2 (Table 2, “model parameter method” column).

**Table 2:** Values of gravitropic (β) and proprioceptive (γ) sensitivities (SI unit) and B determined with *AC* model phenotyping with the “parameter model method” and the “final shape method”, on the whole stem.

	Seedling 1		Seedling 2	
	Parameter method	Final shape method	Parameter method	Final shape method
β	-4.3346e-06	-	-9.6972e-05	-
γ	-7.1651e-07	-	-1.1671e-05	-
β/γ	6.05	4.524	8.05	5.284
L0 (m)	0.43	0.43	0.38	0.38
B	2.12	1.94	3.1	2.01

Values of the B parameter were determined on the stem shape at gravitropic equilibrium: For each of the two poplar seedlings, in order to determine B (Eq 3), the local inclination at gravitropic equilibrium (GSA) vs. the position along the stem (curvilinear abscissa) was fitted with an exponential function (S4 and S5 Figs). Because the steady state shape does not depend on the motor efficiency (but just on the driving), the values of B were computed for data concerning the radially growing zone of the stems (like before) but also from data concerning the whole stems in order to assess if similar driving could operate on the primary and secondary growth zone.

Analysis of the radially growing zone of the stems led to B = 2.26 for seedling 1 and B = 2.21 for seedling (Table 1, “final shape method” column), whereas the analysis of the whole stems led to B = 1.94 for seedling 1 and B = 2.01 for seedling 2 (Table 2, “final shape method” column).

Test of consistency of the two methods for estimating the values of B was performed: Parameter values obtained by the two methods (“model parameter method” and”final shape method”) for the two stems did not significantly differ at the level of 5% for the part undergoing radial growth (P value = 0.33) or for the whole stem (P-value = 0.39), regardless of the method used.

#### *ArC* model

β, γ and ν parameter values of the *ArC* model were determined by linear regression on the tropistic kinematics:Values of parameters (β,γ and ν) and results of the regression statistics of the *ArC* model are given in S4 Table. Linear regression of the *ArC* model was highly significant for all trees and the parameter values were significant for most of the regressions. However, one of the values of the three parameters was not statistically significant at the 5% confidence level for 20% of the trees.

Test of consistency of the estimation of B from the *ArC* and *AC* models was performed: The parameters of the *ArC* model fitted on the data of the anisotropic experiments led to an average value of B = 0.7. B values computed from the parameters of model *AC* obtained from isotropic light experiments were around 2. The use of the *ArC* model parameters thus led to B values about twice as small as the *AC* model parameters.

**Fig 8:**
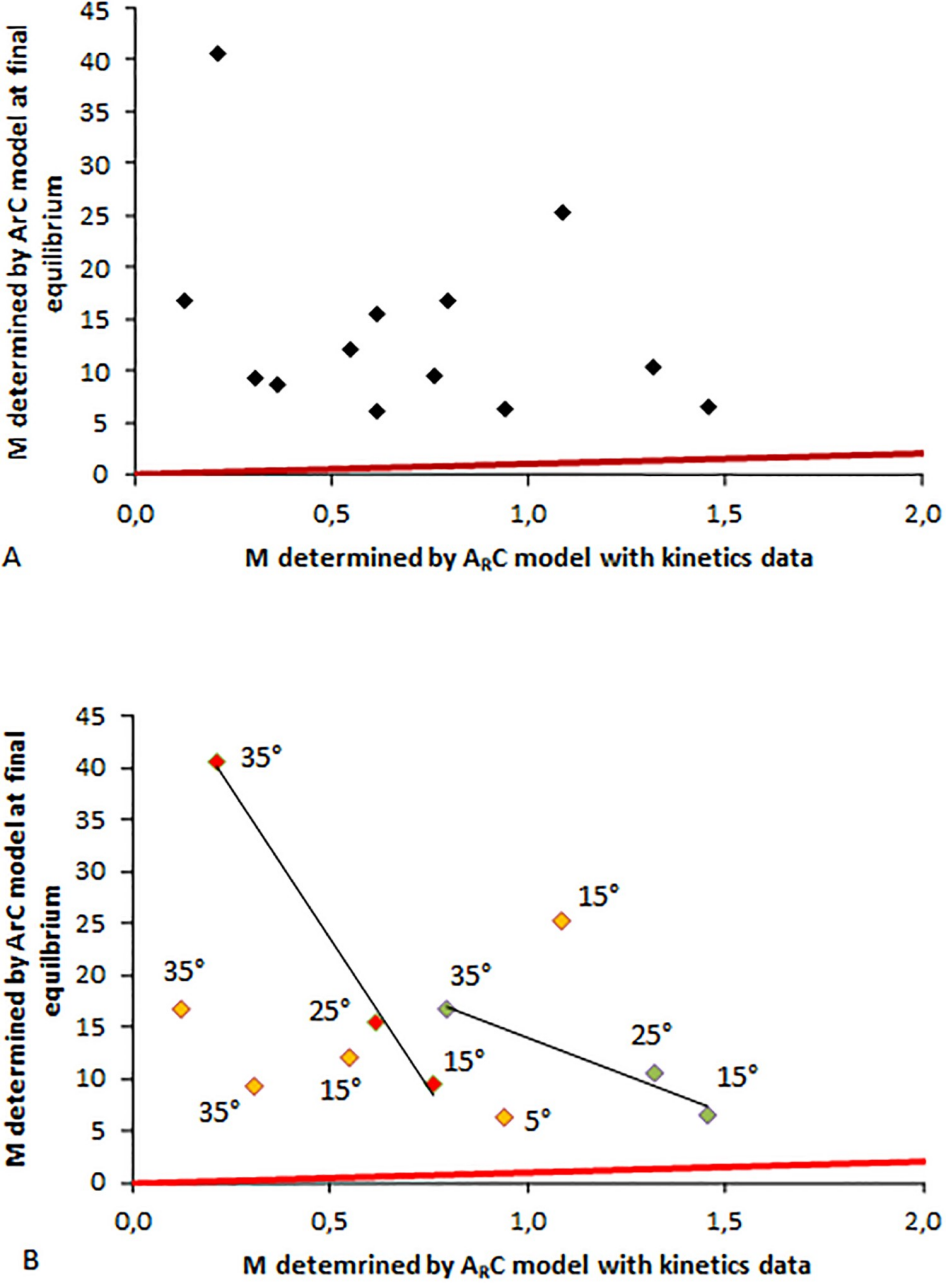
Relationship between M values of poplars computed from shapes at PGSA and M values computed from *ArC* model parameters given by linear regression. A: Whole dataset. B: Split dataset for the different light fluence rates. Green symbols: 15W neon tubes; Orange symbols: 40W neon tubes; Red symbols: 55W neon tubes. Initial tilting angle of the pot is indicated for each piece of data. Solid red line: line 1:1.

A consistency test of the methods for estimating the values of M using the *ArC* model in kinetics or at steady state was performed: The M values obtained from linear regression of the *ArC* model with kinetics data of photogravitropic movements and those obtained from phenotyping stem shape at steady state (PGSA) with the *ArC* model were compared (Fig 8).

When considering the whole dataset (Fig 8A), several remarks can be made. First, the values of M determined from final shapes are always higher than the values of M determined from kinematics data. To find some possible explanations for this discrepancy, we examined the relationship by clustering the data by light fluence rates in the treatments (Fig 8B). Doing so, the correlation coefficients were much higher than the ones obtained using the whole dataset with no clustering. As a general trend, the data obtained for a given lighting for small tilting treatments were the closest to the line 1:1. This result stems from the fact that when designing the *ArC* model, the sine law for both the angular response vs. gravity and vs. the light sources has been simplified to the first (linear) term of the sine Taylor series (see, for example, Equations. 4 and 11 in [3]). This simplification is obviously only accurate for small angles. It is therefore almost not biased concerning the shape prediction by the model in the steady-state form (in general, the angles are moderate) or at the end of the tropistic kinematics, but the bias is greater at the beginning of the kinematics when the angles vs. gravity and/or the light source are high.

As a conclusion for these results on poplar, the determination of B by the *AC* model parameter method or by the final shape method provided good results, indicating that the *AC* model is validated when considering data concerning the pure gravitropic response of the trees. The determination of B by the *ArC* model parameters led to an underestimation of B, very probably due to our tilting device in anisotropic light (see Discussion section).

The determination of M by the *ArC* model parameters obtained by the model parameter method and by the final shape method led to a four-fold discrepancy. This discrepancy can be easily attributed to problems of correlation of explicative variables due to our tilting device in anisotropic light, on the one hand, and to the possible lack of accuracy on the measurement of Ar on final stem shape, on the other. We thus concluded that the *ArC* model, already validated with data on herbaceous plants, could not be invalidated by our data on woody plants. We thus performed a model-assisted phenotyping of stem shape at equilibrium for oak and beech to determine their ratio of gravitropism/proprioception and gravitropism/phototropism sensitivities.

### Model-assisted phenotyping of stem shapes at GSA or PGSA steady state: For poplar, oak and beech

The average values of B and M determined from final shapes are given for each of the three species tested in Table 3, and the Ar and Ap values for M computations are given in S5 Table.

**Table 3:** **Summary of mean values and standard deviation of values of B and M parameters computed from stem shapes at gravitropic and photogravitropic equilibrium, respectively, for three species:** Oak, beech and poplar.

	B	M
Oak	2.89 (± 0.37)	2.27 (± 3.30)
Beech	0.726 (± 0.29)	4.69 (± 0.77)
Poplar	2.23 (± 0.035)	12.51 (± 8.29)

For M values, means were computed including data at different light fluence rates. The standard error is indicated in parentheses.

The values of the B parameter for oak (2.96) and poplar (2.23) are close, indicating similar ratios of sensitivities of gravitropism and proprioception. B appeared to be much smaller for beech (0.73) since beech is less sensitive to proprioception compared to graviperception than poplar and oak.

Oak species had the smallest values of M (M = 1.28) and poplar species the highest (M = 12.5). Beech species had an intermediate value (M = 4.69). We then checked if this variability could be explained by light intensity for oak and poplar.

### Relationship between the equilibrium shape and the initial tilting angle or light fluence rate

**Fig 9:**
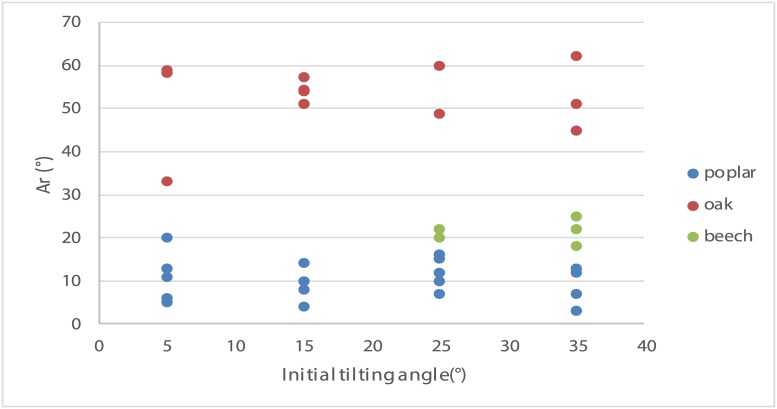
Values of Ar as a function of the initial angle of tilting from the vertical, for poplar, oak and beech. Note: for beech only, Ar values for a tilting angle larger than 20° from the vertical are presented because a tilting angle of less than 20° engendered no movement of the stems.

Fig 9 presents the value of Ar (photogravitropic equilibrium) as a function of the tilting angle for poplar, oak and beech. Variability of Ar is not explained by the initial angle of tilting, in accordance with the prediction of the *ArC* model.

**Fig 10:**
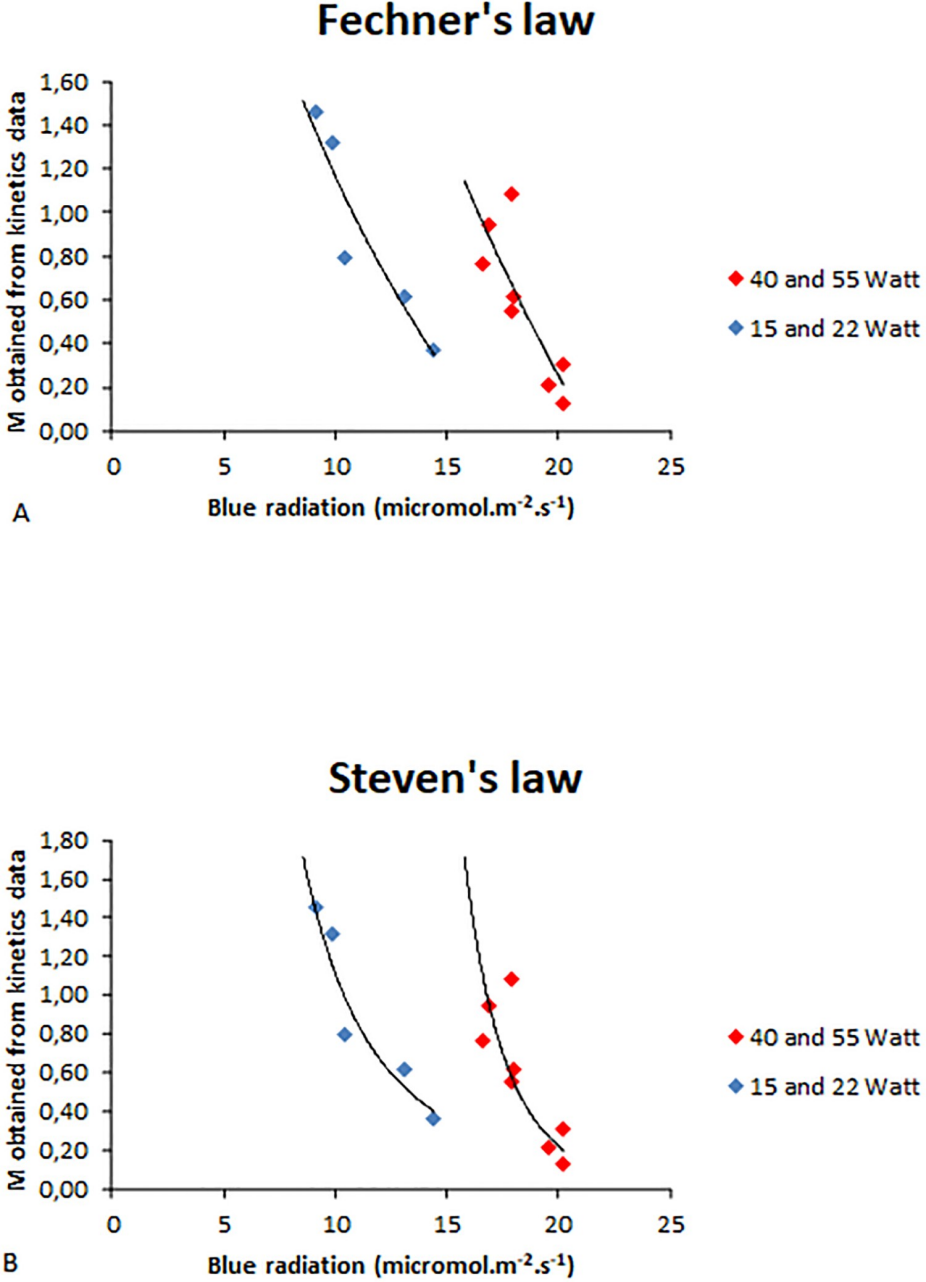
M values (obtained from parameters of the *ArC* model in kinetics) as a function of blue light intensity for poplar. Blue symbols: data obtained with 15W and 22W neon tubes; Red symbols: data obtained with 40W and 55W neon tubes of. One outlier was removed from the plot.
A: Fechner’s law: M values are fitted by a logarithmic function:
Data with 15W and 22W neon tubes: *y* = −2.228 ln(*x*) + 6.2948; *R*^2^ = 0.8788Data with 40W and 55W neon tubes of: *y* = −3.772 ln(*x*) + 11.548; R^2^ = 0.6974B: Steven’s law: M values are fitted by a power function:
Data with 15- and 22-watt neon tubes: *y* = 682.59*x*^−2.789^; R^2^ = 0.9198Data with neon 40- and 55-watt tubes of: *y* = 4*E*10x^−8.645^; R^2^ = 0.7617

Fig 10 presents M values for poplars plotted as a function of light intensity (fluence rate of blue radiation). Data appeared to be divided into two groups, one corresponding to fluence rate values of 15W and 22W, and the other to fluence rate values of 40W and 55W. Each data group was fitted either by a power function (Fig 10A) or by a logarithmic function (Fig 10B), leading in both cases to high correlation coefficients (one outlier was removed from the fit). However, correlation coefficients were higher with fitting by a power function.

**Fig 11:**
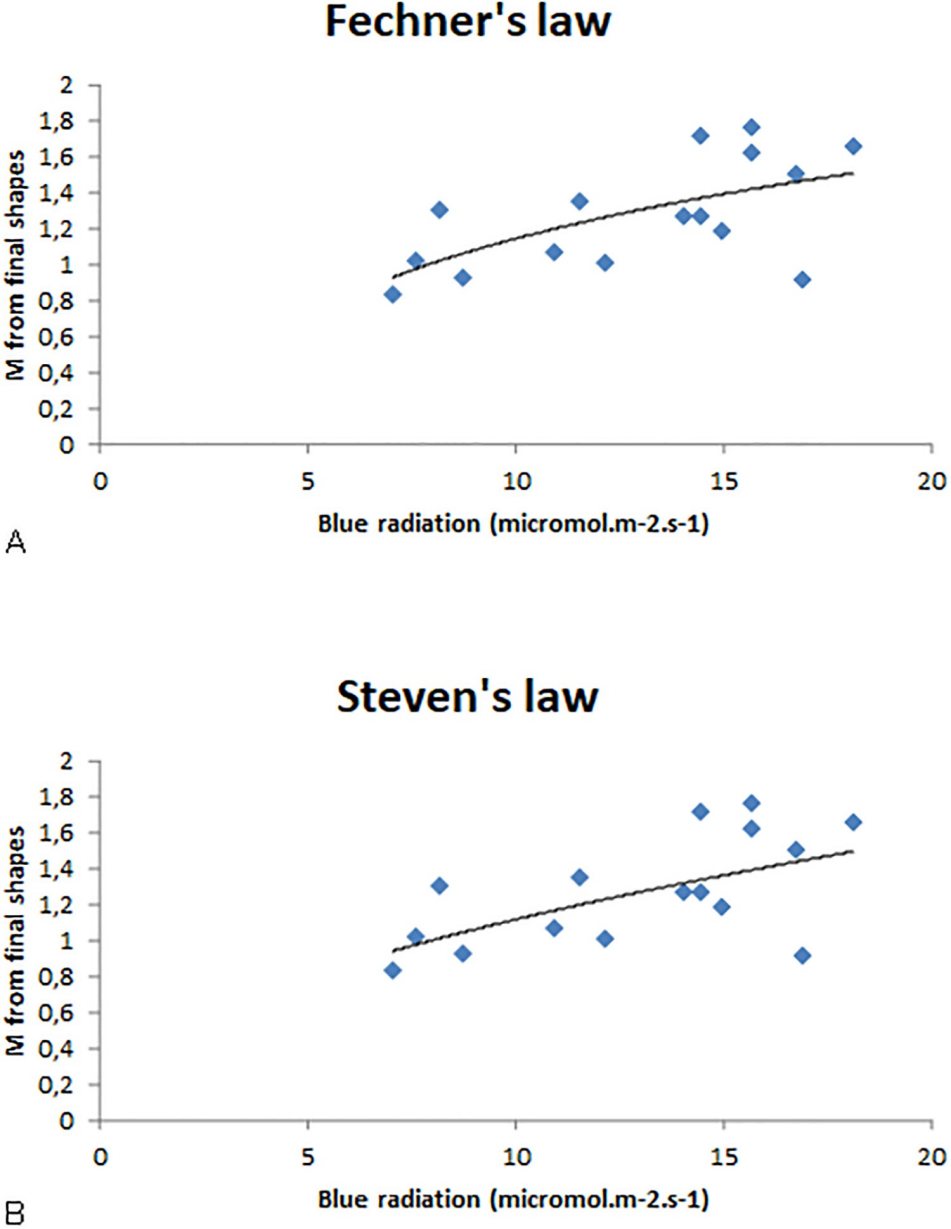
M values as a function of blue light intensity for oak. A: Fechner’s law: M values are fitted by a logarithmic function: *y* = 0.6253 ln(*x*) − 0.2899; R^2^ = 0.2848. B: Steven’s law: M values are fitted by a power function: *y* = 0.287x^0,0.579^; R^2^ = 0.3415. Two outliers were removed from the plots.

Fig 11 presents the M values for oaks as a function of light intensity. The relationship can also be fitted with a logarithmic function (Fig 11A) or with a power function (Fig 11B) (two outliers were removed from the fits). The correlation coefficient of the fit was slightly higher with a power function.

## Discussion

### Characteristics of tropistic movements in the three species tested

This is the first time that gravitropic and phototropic movement have been disentangled and quantitatively analyzed in young trees. In particular, thanks to the isotropic light device, the gravitropic response alone under light conditions could be analyzed.

The three species displayed clear gravi- and phototropistic movements. Poplar displayed a classical tropistic movement consistent with previous reports [19]. However, oak and beech displayed more complex behavior. In oak, the phototropic movement seems to be linked to the rhythmicity of growth, i.e., alternating phases of activity and phases of apparent inactivity of the terminal bud.

Concerning beech, in this study we focused on cases where tropistic movement could be observed. Nevertheless, the cases where no movement could be detected are also interesting. They suggest the existence of a gravitropic threshold in terms of inclination perception for gravitropism, which has, to our knowledge, never been demonstrated so far. Recent works on statoliths and gravisensing [27] reported that “packed grains resting at the bottom of a container full of liquid will not move when the container is inclined unless the inclination becomes higher than a critical angle, typically around 25° for spherical grains”. In herbaceous plants, statoliths have been found not to behave like passive grains: they are subjected to constant active agitation that helps the grains to rearrange when a plant is titled, even at small angles, and to trigger a gravitropic response of the plant [27]. In the case of beech, it might be hypothesized that through evolution, this tree species has lost its function of statolith agitation. This could explain why young beech trees tilted less than 20° do not exhibit any gravitropic response. This hypothesis should now be assessed through microscopic experiments on beech statoliths.

### Validity of the models and accuracy of B and M values

All regressions processed on kinetics data were highly significant, so that the two models (*AC* and *ArC*) explained a significant part of the processes. The determination coefficient was approximately 0.35 on average, which can appear to be not very high. Nevertheless, it must be kept in mind that the curvature is obtained by double spatial derivation of the shape so that the curvature variable is very sensitive to small local variations of the stem shape and to any digitizing noise. In addition, following this double spatial derivation, the curvature rate is obtained by temporal derivation of the curvature values, which also amplify the effect of small variations. It was thus expected that the variables would be noisy.

Overall, the two models seemed to provide interesting insights and could be used to phenotype the plants through the Balance number B and the Motion-pointing number M. As a general trend, it can be observed that B values computed from the *AC* model (using data obtained in isotropic light experiments) by the “model parameter method” or by the “final shape method” are close (around 2).

The discrepancy with the B values obtained by the fitting of the *ArC* models on the kinetics confirmed the recommendation of Bastien et al.: B values should be determined preferentially with data from experimental data of the sole gravitropic movement. This adds to the usefulness of the isotropic light device for any plant that, contrary to coleoptiles, requires light for growth.

The values of M computed by the *ArC* model parameter method are larger than those obtained by the final shape method. We attributed these differences to the correlation of predictive variables due to the design of the bending system in an anisotropic light environment, as explained earlier in the Results section.

It should be noted that the computation of M from Ar and from Ap is far more rapid than from values of sensitivity parameters obtained by linear regression of kinetics data of the tropistic movements. Nevertheless, it should also be observed that the determination of M on final stem shape is fast but quite error-sensitive because a small variation of Ar measurement leads to a rather large vibration of the M value: an error of 2° on Ar measurement leads to an error on the M value of about 15–20%.

Further insight can be gathered here by comparing the 55W and 40W treatment. As explained in the M&M section, they lead to very similar PAR and blue radiation. Moreover, it is better to focus only on the response to small angles of 15° and 5° (small angles are considered to avoid problems with large angles, as explained earlier). The variability on M values (measured as the standard error (SE)) computed at angles of 15° and 5° was 6.8 and 5.2 for poplar and 0.24 and 0.21 for oak, respectively. These values indicate that individual variability is smaller than variability due to the combined effects of individuals and light effects (SE = 8.29 for poplar and SE = 3.3 for oak; see Table 3). Thus, phenotyping M using measurements on shapes at photogravitropic equilibrium is a fast and reliable way to determine the ratio of gravitropic to phototropic sensitivities at small and moderate tilting angles.

Taken all together, it can therefore be concluded that the “final shape method” under isotropic and anisotropic light for the estimation of the value of the Balance number B and the Motion-pointing number M, respectively, is far more rapid and requires less data acquisition and analysis than the “model parameter method”.

### Independence of the photogravitropic equilibrium angle Ar from the tilting angle and dependence of Ar and M values on light intensity

Our results are in accordance with the results of Bastien et al., and contrary to the claim by Galland et al. [8]: the gravitropic and photogravitropic equilibrium is independent of the tilting angle [3, 20].

Relationships were found between Ar (and M) and the light intensity in both poplar and oak but did not make it possible to determine if M was piloted by light intensity following Fechner’s or Steven’s law. This can be easily attributed to the fact that in our experiments, the blue radiation fluence rate varied from 6 to 20 micromol.m^-2^.s^-1^. This approximately corresponds to 1.2 to 4 W.m^-2^, or a range covering a power magnitude of approximately 4, whereas in the studies of Galland on coleoptiles, revisited by Bastien et al. [3], the fluence rate ranged from 1E-10 to 1 W.m^-2^, or a range covering a power magnitude of 10.

### Possible improvements of the experimental device

Several improvements of the experimental conditions to study the photogravitropic responses can be envisioned: since growth is a *sine qua non* condition for tropistic movements, a sufficient quantity of PAR is required during the experiment. In our conditions, both isotropic light and anisotropic light devices provided enough PAR to ensure growth. The anisotropic light has the disadvantage of combining the anisotropic blue light-induced phototropic signal and anisotropic PAR. To circumvent this limit, a possibility could be to add LEDs that generate blue radiation within the isotropic light device. The isotropic device would then ensure photosynthesis and growth under isotropic PAR, and the additional LEDs would provide a good directional light source to trigger the phototropic reaction. This system would make it possible to manipulate the level of blue radiation for a given level of PAR and to efficiently control the direction of blue light and to thus disentangle tropistic and photosynthetic effects of blue light in the regulation of tropistic movements. Most importantly, it would also make it possible to better disentangle the effects of the tilting angle from that of the incipience angle of the blue light tropistic signal.

## Conclusion

The experimental setting combining an isotropic light device and an anisotropic light device used in this study is an original and powerful means to disentangle the gravitropic and phototropic responses of tree seedlings using experiments on Earth ground. The *AC* and *ArC* models have been partially experimentally validated for seedlings and young stems of woody species. Indeed, in these plants, the main stem is sufficiently slender for the motor aspects not to be a limiting factor of tropistic movements, and gravi- and photogravitropic equilibrium can be reached in a few weeks. Then, by combining isotropic and anisotropic light devices and model-assisted phenotyping of the tropistic drivers of the plants, we were able to develop a unique method to compute the Balance number B and the Motion-pointing number M (ratios of sensitivities of gravitropism/proprioception and gravitropism/phototropism, respectively), two intensive quantitative traits that summarized most of the control of the gravitropic and photogravitropic behavior.

To our knowledge, this is the first time that this type of study on tropisms has been conducted on woody species. The phenotyping of the Balance and Motion-pointing numbers B and M on the stem shapes at gravi- or photogravitropic equilibrium (steady state) was found to be the most convenient approach since it requires less data, and both data measurement and analysis are rapid. This offers the possibility of phenotyping a larger number of stems, which is a requirement for comparing a large number of species or individuals.

Using this method, we were able to quantitatively discriminate three hardwood species contrasted by their mode of growth, habit and light requirements: poplar, oak and beech, through their B and M values (although more replicates are needed for a full assessment of these differences). It can also be suggested that the driving of tropism in the primary growth zone (motorized by differential primary elongation) and in the secondary growth zone (motorized by reaction wood formation) may be the same.

Our method was tested on very young stems. The extension of this work to larger trees (eventually to adult trees) is not straightforward. In addition to the obvious problem of size and duration of experiments, the equilibrium might not be reached in one growing season because of movement limitations due to the combination of the (large) bending resistance of the trunk and the (small) growth in girth [18]. It is thus likely that the coupling of the *AC* and *ArC* driving models with process-based models of the tropistic motor [14–15] will be necessary. Nevertheless, it is likely that the control by the photo- and gravitropic sensitivities is genetically encoded and constant throughout the life of a tree, so that phenotyping of juvenile trees can also be relevant for their later stages [28]. Bastien et al. [3] reported values of B between 2.5 and 3.5 for two-year-old poplars (deltoides*nigra). In this outdoor experiment [19], the gravitropic stimuli was combined and collinear with the phototropic stimulus. It is then logical that the B values calculated using the *AC* model (without a proper account of phototropism) were higher than the ones found for pure gravitropism in this experiment (B = 2.3). However, the possible effect of phototropic stimulus in this experiment was minimized through a northwise leaning of the trees, and the use of a genotype considered by poplar practitioners as not very light-sensitive. Additionally, in the present study, poplar was found to be much more sensitive to gravitropism than to phototropism (M = 12), so that this overestimation should be small. In fact, the two values for poplar seedlings and two-year-old saplings are in the same range and very different from what was found for oak (B = 0.7), giving some experimental credit to the hypothesis that the values of B do not depend on the stage of the plant but primarily on its genotype.

Aside from a direct B-M phenotyping of adult trees, an alternative way could be to assess the relationship between the B-M values of very young individuals and the general habit of the mature tree. For example, since B (ratio of gravitropism and proprioception sensitivities) controls the degree of curving of an axis, it would be interesting to measure B on seedlings of a large number of genotypes (contrasted by their the more or less curved shape of their axes in adult trees), and to then check if B variability would explain the variability of axis shape (from straight to curved). Indeed, such juvenile*adult correlation on regulatory traits usually display increased genetic heritability and, consequently, are powerful tools for early selection [29].

As another example, our results indicated that poplar may exhibit the highest gravitropism/phototropism ratio and oak the smallest. Moreover, mature poplar exhibits a more erect habit than oak. It may thus be hypothesized that the ratio of gravitropism/phototropism is a significant determinant of natural tree habit.

It is too early to draw statistically-substantiated definitive conclusions on these last topics from these first experiments. Nevertheless, our work provides illustrations of the insights that our approach can provide and raises hypotheses linking tropistic sensitivities and tree habit. These hypotheses should now be assessed on a large number of individuals, as well as on more species and genotypes.

## Supporting information

S1 Fig Spectra provided by 15W, 22W, 40W and 55W neon tubes. (TIF)

S2 Fig Scheme presenting the computation of the angle of the stem from the vertical at the photogravitropic equilibrium (Ar) angle, angle of light direction from the vertical (Ap) and angle between the apex and the light direction (App). A: For poplar and oak, Ar was measured at the tip of the stem. C: For beech, because the annual shoot always remained curved (no tropistic reaction), Ar was measured just below the base of the annual shoot. Successive shapes of a titled tree in an anisotropic light environment are represented in colors. The annual shoot is represented by a solid line.
(TIF)

S3 Fig PAR as a function of the tilting angle of the light source from the vertical for 15W, 22W, 40W and 55W neon tubes. (TIF)

S4 Fig Local inclination vs. curvilinear abscissa along the whole stem, fitted by an exponential function for two poplar seedlings tilted 35° from the vertical in an isotropic light environment. A: Poplar seedling 1. B: Poplar seedling 2.
(TIF)

S5 Fig Local inclination vs. curvilinear abscissa along the radially growing zone fitted by an exponential function for two poplar seedlings tilted 35° from the vertical in an isotropic light environment. A: Poplar seedling 1. B: Poplar seedling 2.
(TIF)

S1 Table Values of PAR and blue radiation measured in the anisotropic light conditions for the different neon tubes and the different tilting angles. PAR was measured on the side of the stem facing the neon tubes. PARopp was measured on the side of the stem opposite the neon tubes. Power values of neon tubes are given in watts, tilting values are given in degrees, PAR and blue radiation values are given in micromol.m^-2^.s^-1^. “Nb tube” refers to the number of neon tubes (two were turned on in experiments with oak and beech; three were turned on in experiments with poplar).
(DOCX)

S2 Table Average daily diameter growth rate of stems at 10 cm from stem base. Means were computed per level of fluence rate without taking inclination into account (A); or computed by level of inclination without taking fluence rate into account (B). Daily growth rate is given in mm.day^-1^.
(DOCX)

S3 Table Values of sensitivities to gravitropism (β) and proprioception (γ) and statistics of the model, obtained from linear regression analysis of kinetics data of gravitropic movement by the *AC* model on the radially growing zone and on the whole stem. (DOCX)

S4 Table Parameters of sensitivities to gravitropism (β), phototropism (ν) and proprioception (γ), their P values and model statistics obtained with *ArC* model. Values were obtained from linear regression of the *ArC* model with kinetics data of tropistic movements of young poplars subjected to tilting with an anisotropic light device (light came from the lower side of the plant). Values of parameters are given in SI units. α is the initial angle of tilting from the vertical. Ap is the angle between the stem apex and the light direction.
(DOCX)

S5 Table Values of initial tilting angle, power of neon tubes, Ar and Ap for poplar, oak and beech. Angle values are given in degrees; power of neon tubes are given in watts.
(DOCX)
