# Supplementary figures and images for: A method for the quantification of phototropic and gravitropic sensitivities of plants combining an original experimental device with model-assisted phenotyping: Exploratory test of the method on three hardwood tree species
Source: PLoS One. 2019 Jan 25;14(1):e0209973. doi: 10.1371/journal.pone.0209973 (PMC6347157; doi:10.1371/journal.pone.0209973)

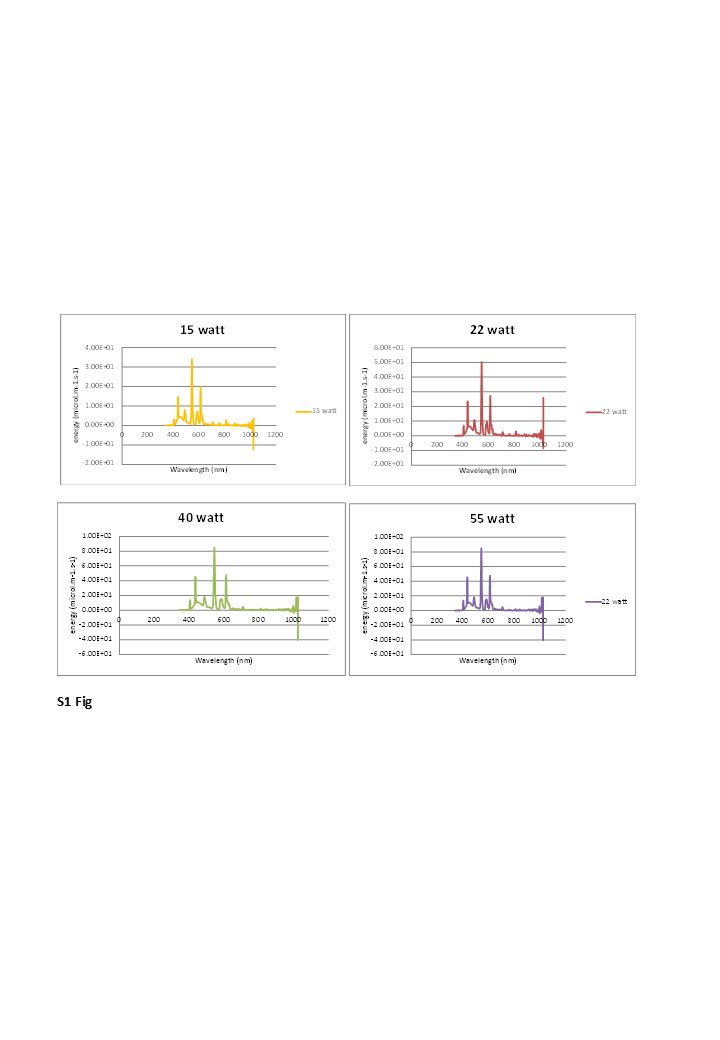

Supplement: S1 Fig — (TIF) [file pone.0209973.s001.tif]

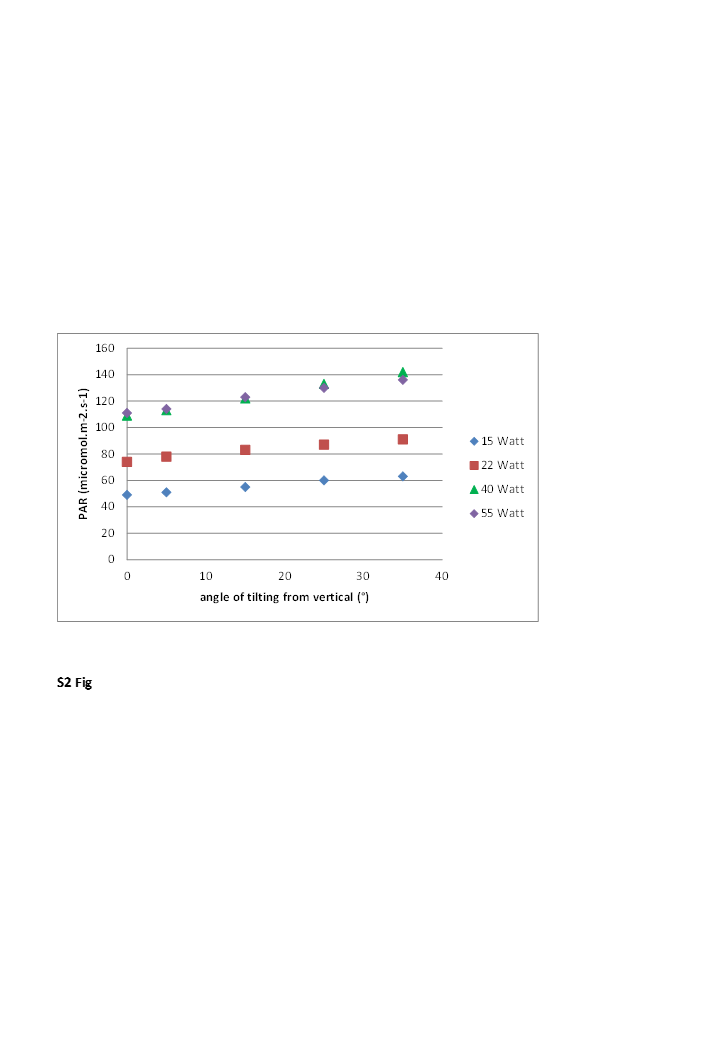

Supplement: S2 Fig — A: For poplar and oak, Ar was measured at the tip of the stem. C: For beech, because the annual shoot always remained curved (no tropistic reaction), Ar was measured just below the base of the annual shoot. Successive shapes of a titled tree in an anisotropic light environment are represented in colors. The annual shoot is represented by a solid line. (TIF) [file pone.0209973.s002.tif]

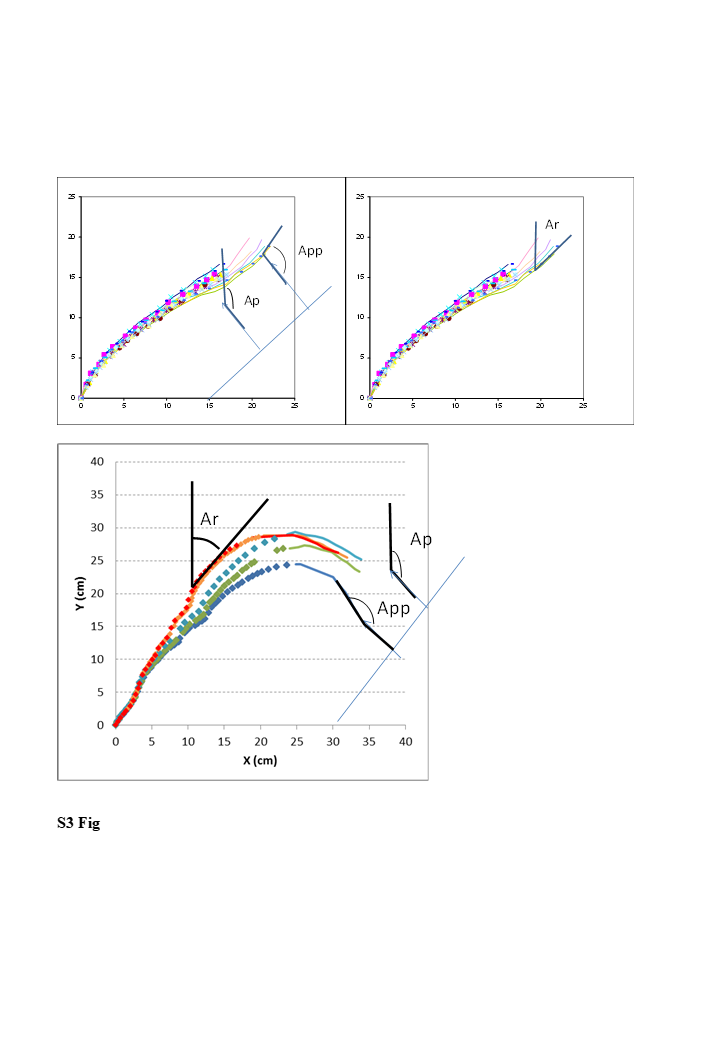

Supplement: S3 Fig — (TIF) [file pone.0209973.s003.tif]

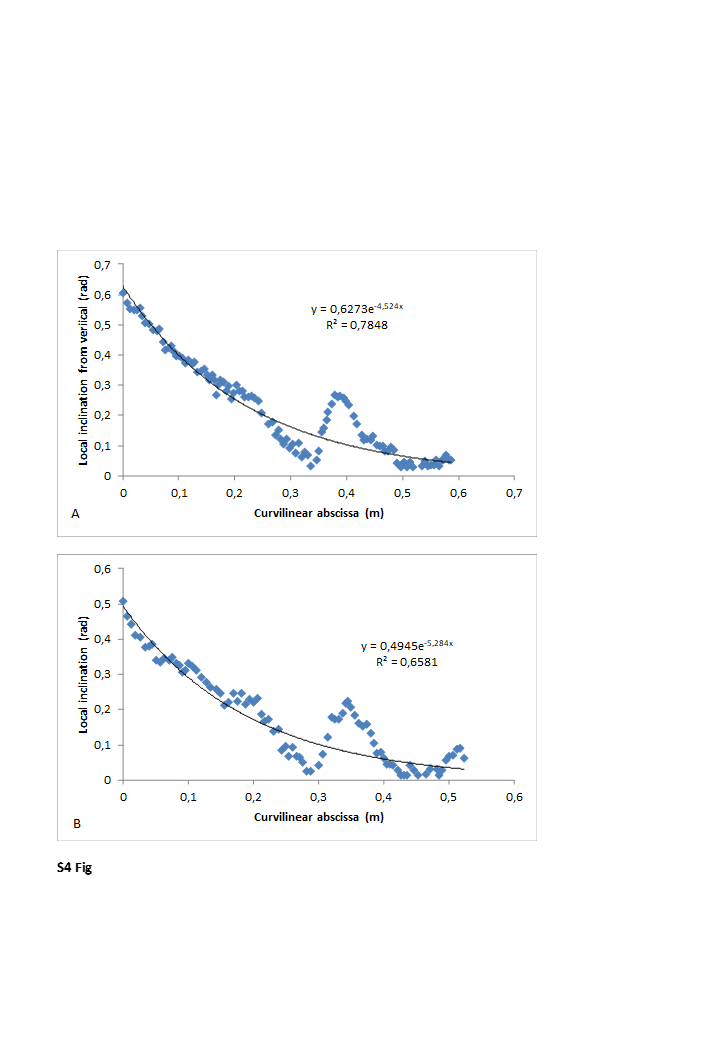

Supplement: S4 Fig — A: Poplar seedling 1. B: Poplar seedling 2. (TIF) [file pone.0209973.s004.tif]

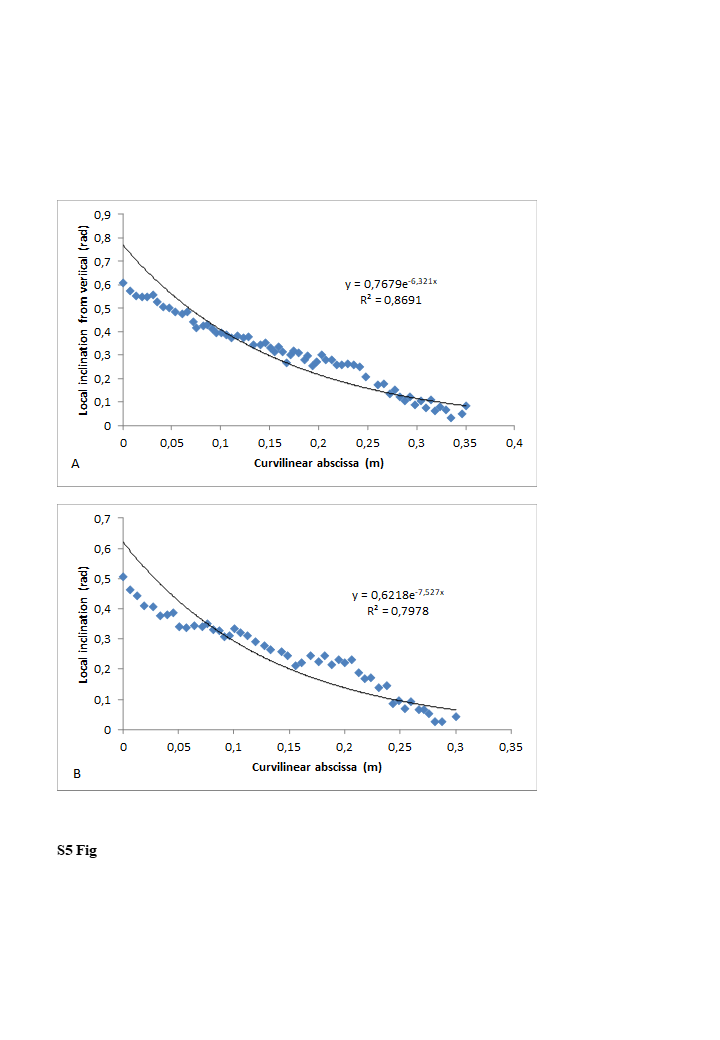

Supplement: S5 Fig — A: Poplar seedling 1. B: Poplar seedling 2. (TIF) [file pone.0209973.s005.tif]
