# Supplementary material for: A method for the quantification of phototropic and gravitropic sensitivities of plants combining an original experimental device with model-assisted phenotyping: Exploratory test of the method on three hardwood tree species
Source: PLoS One. 2019 Jan 25;14(1):e0209973. doi: 10.1371/journal.pone.0209973 (PMC6347157; doi:10.1371/journal.pone.0209973)
Supplement: S1 Table — PAR was measured on the side of the stem facing the neon tubes. PARopp was measured on the side of the stem opposite the neon tubes. Power values of neon tubes are given in watts, tilting values are given in degrees, PAR and blue radiation values are given in micromol.m-2.s-1. “Nb tube” refers to the number of neon tubes (two were turned on in experiments with oak and beech; three were turned on in experiments with poplar). (DOCX) [file pone.0209973.s006.docx]

| **puissance** | **tilting** | **nb neon** | **PAR** | **PARopp** | **blue** |
| --- | --- | --- | --- | --- | --- |
| 15 | 5 | 2 | 51 | 2 | 6,97 |
| 15 | 15 | 2 | 55 | 2 | 7,50 |
| 15 | 25 | 2 | 60 | 2 | 8,23 |
| 15 | 35 | 2 | 63 | 2 | 8,66 |
| 22 | 5 | 2 | 78 | 2 | 10,58 |
| 22 | 15 | 2 | 83 | 2 | 11,26 |
| 22 | 25 | 2 | 87 | 2 | 11,80 |
| 22 | 35 | 2 | 91 | 2 | 12,37 |
| 40 | 5 | 2 | 113 | 2 | 15,27 |
| 40 | 15 | 2 | 122 | 3 | 15,51 |
| 40 | 25 | 2 | 133 | 3 | 16,95 |
| 40 | 35 | 2 | 142 | 3 | 18,00 |
| 55 | 5 | 2 | 114 | 5 | 15,78 |
| 55 | 15 | 2 | 123 | 5 | 16,63 |
| 55 | 25 | 2 | 130 | 4 | 17,81 |
| 55 | 35 | 2 | 136 | 4 | 19,54 |
| 15 | 5 | 3 | 62 | 3 | 8,56 |
| 15 | 15 | 3 | 66 | 3 | 9,09 |
| 15 | 25 | 3 | 72 | 3 | 9,90 |
| 15 | 35 | 3 | 76 | 3 | 10,41 |
| 22 | 5 | 3 | 88 | 3 | 11,66 |
| 22 | 15 | 3 | 93 | 3 | 12,34 |
| 22 | 25 | 3 | 99 | 3 | 13,12 |
| 22 | 35 | 3 | 108 | 3 | 14,41 |
| 40 | 5 | 3 | 132 | 3 | 16,89 |
| 40 | 15 | 3 | 140 | 3 | 17,84 |
| 40 | 25 | 3 | 149 | 3 | 18,85 |
| 40 | 35 | 3 | 160 | 3 | 20,20 |
| 55 | 5 | 3 | 129 | 6 | 15,78 |
| 55 | 15 | 3 | 136 | 5 | 16,63 |
| 55 | 25 | 3 | 147 | 5 | 17,98 |
| 55 | 35 | 3 | 160 | 4 | 19,54 |

S1 Table
