# Supplementary material for: A method for the quantification of phototropic and gravitropic sensitivities of plants combining an original experimental device with model-assisted phenotyping: Exploratory test of the method on three hardwood tree species
Source: PLoS One. 2019 Jan 25;14(1):e0209973. doi: 10.1371/journal.pone.0209973 (PMC6347157; doi:10.1371/journal.pone.0209973)
Supplement: S3 Table — (DOCX) [file pone.0209973.s008.docx]

|  | Parameters values | |  | P of parameters | | Model statistics | |  |
| --- | --- | --- | --- | --- | --- | --- | --- | --- |
|  | intercept | β | γ | β | γ | P | F | R^2^ |
| seedling 1 (radially growing zone) | -1.75e-06 | -5.8484e-06 | -8.5109e-07 | 1.9406e-12 | 5.6575e-36 | 9.29e-40 | 109 | 0.308 |
| seedling 2 (radially growing zone) | -5.5662e-06 | -2.2874e-05 | -2.8895e-06 | 3.1019e-16 | 1.1902e-20 | 2.94e-29 | 73.7 | 0.202 |
| seedling 1 (whole stem) | 4.8117e-07 | -4.3346e-06 | -7.1651e-07 | 0.00048959 | 1.1236e-07 | 7.17e-07 | 15.1 | 0.118 |
| seedling 2 (whole stem) | -1.7002e-05 | -9.6972e-05 | -1.1671e-05 | 1.8779e-114 | 3.7453e-117 | 5.47e-123 | 1.33e+03 | 0.924 |

S3 Table
