# Supplementary material for: A method for the quantification of phototropic and gravitropic sensitivities of plants combining an original experimental device with model-assisted phenotyping: Exploratory test of the method on three hardwood tree species
Source: PLoS One. 2019 Jan 25;14(1):e0209973. doi: 10.1371/journal.pone.0209973 (PMC6347157; doi:10.1371/journal.pone.0209973)
Supplement: S4 Table — Values were obtained from linear regression of the ArC model with kinetics data of tropistic movements of young poplars subjected to tilting with an anisotropic light device (light came from the lower side of the plant). Values of parameters are given in SI units. α is the initial angle of tilting from the vertical. Ap is the angle between the stem apex and the light direction. (DOCX) [file pone.0209973.s009.docx]

| α (°) | (Ap)(°) | Neon tubes | Parameters values (SI units) | | | P value of parameters | |  | Model statistics | |  |
| --- | --- | --- | --- | --- | --- | --- | --- | --- | --- | --- | --- |
|  |  | power (Watt) | β | γ | ν | β | γ | ν | P | F | R^2^ |
| -15 | -105 | 15 | -7,43E-05 | -4,03E-07 | -5,10E-05 | 1.703e-49 | 0.20443 | 1.8739e-25 | 8.5e-95 | 299 | 0.727 |
| -35 | -125 | 15 | -5,49E-05 | -1,94E-06 | -6,91E-05 | 5.9665e-25 | 1.3824e-08 | 4.7147e-30 | 2.32e-114 | 451 | 0.809 |
| -25 | -115 | 15 | -5,45E-05 | -1,38E-06 | -4,14E-05 | 1.0883e-68 | 3.4362e-08 | 3.1124e-40 | 1.18e-167 | 803 | 0.859 |
| -5 | -95 | 15 | -3,00E-06 | -5,91E-06 | -2,69E-06 | 0.57191 | 2.4676e-22 | 0.37633 | 1.67e-46 | 100 | 0.471 |
| -5 | -95 | 22 | -6,16E-06 | -2,73E-06 | -7,62E-06 | 0.11838 | 1.1599e-07 | 0.0057012 | 1.9e-15 | 26.5 | 0.189 |
| -15 | -105 | 22 | -7,73E-05 | -8,99E-07 | -2,27E-05 | 6.6625e-42 | 0.0045699 | 9.7204e-11 | 1.32e-62 | 166 | 0.632 |
| -35 | -125 | 22 | -1,96E-05 | -2,82E-06 | -5,38E-05 | 0.0008137 | 3.6982e-09 | 4.7204e-09 | 4.23e-29 | 58.6 | 0.398 |
| -25 | -115 | 22 | -2,09E-05 | -1,13E-06 | -3,41E-05 | 1.0622e-09 | 2.2077e-05 | 2.2161e-13 | 1.57e-34 | 67.4 | 0.362 |
| -15 | -105 | 40 | -3,10E-05 | -1,39E-06 | -2,85E-05 | 6.8675e-36 | 7.1508e-10 | 5.9175e-26 | 1.97e-63 | 139 | 0.482 |
| -5 | -95 | 40 | 1,50E-05 | -1,59E-06 | -1,45E-05 | 5.632e-08 | 4.6819e-09 | 4.085e-05 | 2.08e-31 | 56.3 | 0.25 |
| -25 | -115 | 40 | -1,34E-05 | -2,77E-06 | -6,88E-06 | 0.042269 | 1.2452e-06 | 0.37396 | 2.57e-13 | 22.7 | 0.18 |
| -35 | -125 | 40 | -2,17E-05 | -2,26E-06 | -7,04E-05 | 4.7545e-10 | 4.0189e-13 | 1.3368e-37 | 3.39e-101 | 375 | 0.79 |
| -25 | -115 | 40 | 9,33E-05 | -5,30E-06 | -1,74E-04 | 2.0877e-12 | 1.202e-16 | 3.07e-31 | 6.57e-68 | 184 | 0.647 |
| -15 | -105 | 40 | -7,04E-05 | -6,82E-07 | -1,28E-04 | 2.0558e-33 | 0.058247 | 4.641e-55 | 3.09e-85 | 359 | 0.828 |
| -5 | -95 | 40 | -2,75E-05 | -4,91E-08 | -2,93E-05 | 4.7485e-07 | 0.92445 | 5.7746e-08 | 5.37e-15 | 26.2 | 0.219 |
| -35 | -125 | 40 | -6,86E-06 | -1,14E-06 | -5,37E-05 | 0.039908 | 5.872e-05 | 5.6067e-32 | 1.5e-54 | 117 | 0.467 |
| -15 | -105 | 55 | -1,66E-05 | -1,17E-06 | -2,18E-05 | 8.1207e-07 | 0.0090806 | 0.00033089 | 1.06e-10 | 18 | 0.166 |
| -25 | -115 | 55 | -2,09E-05 | -1,13E-06 | -3,41E-05 | 1.0622e-09 | 2.2077e-05 | 2.2161e-13 | 1.57e-34 | 67.4 | 0.362 |
| -5 | -95 | 55 | -3,10E-05 | -6,94E-06 | -4,47E-07 | 2.0604e-18 | 3.1145e-80 | 0.78592 | 8.5e-167 | 1150 | 0.918 |
| -35 | -125 | 55 | -1,74E-05 | -1,27E-06 | -8,22E-05 | 3.4811e-05 | 0.0022705 | 2.6874e-35 | 7.55e-65 | 190 | 0.69 |

S4 Table
