# Supplementary material for: A method for the quantification of phototropic and gravitropic sensitivities of plants combining an original experimental device with model-assisted phenotyping: Exploratory test of the method on three hardwood tree species
Source: PLoS One. 2019 Jan 25;14(1):e0209973. doi: 10.1371/journal.pone.0209973 (PMC6347157; doi:10.1371/journal.pone.0209973)
Supplement: S5 Table — Angle values are given in degrees; power of neon tubes are given in watts. (DOCX) [file pone.0209973.s010.docx]

| species | angle of tilting (°) | neon power (Watt) | Ar (°) | Ap (°) |
| --- | --- | --- | --- | --- |
| oak | 5 | 15 | -59 | -95 |
| oak | 15 | 15 | -54 | -105 |
| oak | 25 | 15 | -60 | -115 |
| oak | 35 | 15 | -45 | -125 |
| oak | 15 | 22 | -51 | -105 |
| oak | 25 | 22 | -49 | -115 |
| oak | 35 | 22 | -62 | -125 |
| oak | 35 | 40 | -47 | -125 |
| oak | 25 | 40 | -60 | -115 |
| oak | 15 | 40 | -38 | -105 |
| oak | 5 | 40 | -42 | -95 |
| oak | 5 | 40 | -35 | -95 |
| oak | 15 | 40 | -40 | -105 |
| oak | 5 | 55 | -42 | -95 |
| oak | 15 | 55 | -48 | -105 |
| oak | 35 | 55 | -50 | -125 |
| poplar | 5 | 22 | -20 | -95 |
| poplar | 15 | 22 | -14 | -105 |
| poplar | 35 | 22 | -13 | -125 |
| poplar | 25 | 22 | -16 | -115 |
| poplar | 15 | 40 | -4 | -105 |
| poplar | 5 | 40 | -5 | -95 |
| poplar | 25 | 40 | -12 | -115 |
| poplar | 35 | 40 | -12 | -125 |
| poplar | 25 | 40 | -15 | -115 |
| poplar | 15 | 40 | -8 | -105 |
| poplar | 5 | 40 | -13 | -95 |
| poplar | 35 | 40 | -7 | -125 |
| poplar | 15 | 55 | -10 | -105 |
| poplar | 25 | 55 | -7 | -115 |
| poplar | 5 | 55 | -11 | -95 |
| poplar | 35 | 55 | -3 | -125 |
| poplar | 15 | 15 | -14 | -105 |
| poplar | 35 | 15 | -7 | -125 |
| poplar | 25 | 15 | -10 | -115 |
| poplar | 5 | 15 | -6 | -95 |
| beech | 25 | 22 | -22 | -115 |
| beech | 25 | 40 | -20 | -115 |
| beech | 35 | 22 | -25 | -125 |
| beech | 35 | 40 | -25 | -125 |
| beech | 35 | 15 | -22 | -125 |
| beech | 35 | 22 | -18 | -125 |
| beech | 35 | 40 | -25 | -125 |
| beech | 35 | 55 | -18 | -125 |

S5 Table
